# Genotype x environment interaction in cassava multi-environment trials via analytic factor

**DOI:** 10.1371/journal.pone.0315370

**Published:** 2024-12-09

**Authors:** Juraci Souza Sampaio Filho, Isadora Cristina Martins Oliveira, Maria Marta Pastina, Marcos de Souza Campos, Eder Jorge de Oliveira

**Affiliations:** 1 Federal University of Recôncavo da Bahia, Centro de Ciências Agrárias, Ambientais e Biológicas, Cruz das Almas, Bahia, Brazil; 2 Embrapa Milho e Sorgo, Sete Lagoas, MG, Brazil; 3 Embrapa Mandioca e Fruticultura, Nugene, Cruz das Almas, Bahia, Brazil; KGUT: Graduate University of Advanced Technology, ISLAMIC REPUBLIC OF IRAN

## Abstract

The variability in genetic variance and covariance due to genotype × environment interaction (G×E) can hinder genotype selection accuracy, especially for complex traits. This study analyzed G×E interactions in cassava to identify stable, high-performing genotypes and predict agronomic performance in untested environments using factor analytic multiplicative mixed models (FAMM) within multi-environment trials (METs). We evaluated 22 cassava genotypes for fresh root yield (FRY), dry root yield (DRY), shoot yield (ShY), and dry matter content (DMC) across 55 Brazilian environments. FAMM was applied to estimate genetic values and environmental loads, revealing significant genetic variance, especially for FRY (0.16–0.92) and broad-sense heritability ($\hat{H}²$) above 0.70 in advanced yield trials. In joint analyses, analytic factor *FA*_4_ explained over 88% of genetic variation for all traits despite high G×E and data imbalance. Positive genetic correlations were found between environments for ShY and DRY (0.99 and 1.0, respectively), while FRY and DMC showed negative correlations (-0.82 and -0.95). Latent regression analysis identified hybrids adaptable to a range of environments, as well as genotypes suited to specific conditions. Moderate correlations between environmental covariables (rainfall, altitude, solar radiation) and FA model loadings suggest these factors contribute to high G×E interactions, notably for FRY. The FAMM model provided a robust approach to G×E analysis in cassava, yielding practical insights for breeding programs.

## 1. Introduction

Cassava (*Manihot esculenta* Crantz) is a globally cultivated crop and plays a crucial role in ensuring food security. Its roots are used in the production of a wide range of industrialized products, making them economically significant for numerous countries [1,2]. Brazilian production of cassava represents approximately 10.0% of global production, being cultivated across 1.19 million hectares, spanning from the North to the South of the country. The cultivation across different biomass exposes cassava to adverse and contrasting climatic and soil conditions, being highly influenced by the environment [3]. The plants respond to a multitude of environmental signals, both biotic and abiotic, and can generate different responses to environmental conditions. This phenomenon, known as genotype-environment (G×E) interaction, poses a challenge in the selection process [4,5].

The G×E interaction refers to the way in which different genotypes (genetic makeup) of a species respond to various environmental conditions. This variation can make it challenging to select genotypes that perform consistently across all environments, as their growth and yield can be highly influenced by several environmental factors. Therefore, new approaches that effectively capture these interactions can help in the selection of the best-performing clones evaluated over different years, environments, and growing seasons.

To address the adverse impacts of genotype-environment interactions, notably the inconsistency in agronomic performance observed over years in the targeted cultivation environments due to varying environmental stimuli, cassava breeding programs in Brazil undertake uniform yield trials (UYT). These trials gather agronomic data to recommend new cassava varieties, employing larger plots (consisting of 40 to 60 plants), greater number of replications, and multiple cultivation sites. Regional comparative tests are typically conducted in parallel with UYT, evaluating commercial varieties and new cassava clones that are nearing commercial release. These evaluations are performed over several years in multi-environment trials (METs), with the goal of evaluating how different environments affect the performance of the genotypes. METs help breeders determine which genotypes are adaptable and stable across various conditions.

METs are characterized by a large number of environments and an imbalance of tests within and between locations. This imbalance arises from the removal of unproductive clones throughout the selection process and their replacement with promising clones. Additionally, the loss of experimental plots further complicates the joint analysis of METs. However, METs provide an opportunity to observe and quantify phenotypic expression levels in response to environmental variations, such as different years and cultivation locations. In this study, the gap lies in the need to better understand the complexities of G×E interactions and their impact on genotype selection, particularly in the context of cassava breeding.

In MET trials, the genetic and environmental effects can be partitioned, allowing for the quantification of each factor’s contribution (G + G×E), thereby facilitating the exploration of the beneficial effects of G×E interaction. Therefore, METs enable the assessment of genotype adaptability to general or specific environments and the evaluation of yield stability across different target environments. These parameters are essential for recommending new cassava clones.

The investigation of G×E interaction involves considering the heterogeneity of genetic variance and covariance across different environments. Genetic variance refers to the variability in a trait that is due to genetic differences among individuals, while covariance is a measure of how two variables change together, in this case, how genetic factors interact with environmental factors. Similar environments tend to elicit similar responses in genotypes, leading to strong genetic correlations [4,6,7]. However, this poses challenges when fitting models to explore the effects of G×E interaction, as it increases the number of estimated parameters [8]. Mixed models offer an alternative approach for analyzing MET assays, as they provide more information and flexibility in estimating variance components and identifying genetic and environmental parameters that are closely related to genotype performance. This aids breeders in making decisions regarding the recommendation of new varieties [9–11].

The use of basic variance component models has been found insufficient in modeling G×E interactions, as they overlook the covariances between genetic and non-genetic effects across diverse environments. In the mixed model approach, the genetic variance and covariance matrix (VCOV) is adjusted to model the genetic and residual correlations between environments. The residual correlation refers to the unexplained variation in the data after accounting for the factors included in the model The unstructured matrix represents the most complex VCOV structure, accounting for the heterogeneity of variations between environments and all possible specific covariances between pairs of environments [12]. In case of METs with large number of environments and genotypes tested, model convergence becomes challenging due to the high number of estimated parameters. Therefore, a flexible model that reduces the number of estimated parameters is often required [10]. As a result, many genetic improvement programs have adopted the mixed model approach, specifically the multiplicative mixed model known as the analytic factor (FA) model [11,13–15].

In contrast to simpler models, the FA mixed model offers several advantages. First, it allows the use of unstructured models, enabling the estimation of variances and covariances associated with G×E in a parsimonious manner. Second, it facilitates the estimation of genotypic and environmental coefficients, including loads and factor scores, respectively. Third, by utilizing best linear unbiased predictors (BLUPs), it enables the estimation of genotypic effects that are correlated across multiple trials. Fourth, it identifies the presence of residual variance heterogeneity in non-orthogonal experiments (utilizing an unstructured type VCOV matrix) and accounts for the effect of genotypes within environments with limited variance parameters, even with a high number of genotypes and environments. Finally, it combines multiple regression and principal component analysis in a single model, accommodating unbalanced data and allowing for selection in untested environments [10,11,13–15]. Furthermore, the FA model facilitates graphical representations through multiple latent regressions and heat maps for visualizing genetic correlations between pairs of environments [15,16].

Although several studies have highlighted the importance and advantages of exploring G×E using the FA model [16–19], this approach has yet to be explored in cassava breeding programs. On the other hand, most quantitative data from MET trials for cassava are unbalanced, exhibit high environmental variance, and show correlations between trials. These analyses can benefit from capturing the VCOV matrix using FA models, which increase the precision and efficiency of selection, even in untested environments. Additionally, incorporating environmental data in the study of G×E interactions generally enhance the robustness and validity of factor analysis results, as environmental data play a critical role by providing context, aiding interpretation, and controlling for confounding variables. Moreover, incorporating environmental covariates enables the prediction of common latent factors with observable GxE interactions [12,20]. Therefore, FA models offer numerous advantages for routine cultivar selection activities in cassava breeding programs.

The hypothesis articulation involves the use of FA models to better understand and predict the interaction between genotype and environment, improving cassava breeding programs through more accurate and efficient selection processes. Therefore, the objectives of this study were: 1) to investigate G×E in historical data from the cassava breeding program at Embrapa, focusing on the adaptability and stability of genotypes using the FA model; 2) to select stable and adapted genotypes with genetic gains for four agronomic variables of interest, namely fresh root yield (FRY), dry root yield (DRY), shoot yield (ShY), and dry matter content (DMC), in target environments; 3) to predict the performance of genotypes in environments where they were not evaluated in the METs; 4) to identify the environmental covariates that best explain G×E and the genetic correlations between environments in the cassava breeding program tests, and 5) Compare the ability to capture G×E interactions and the model fit of the FA model with the AMMI and GGEbiplot methods.

## 2. Material and methods

### 2.1. Genetic material and experimental design

The dataset used in this study consisted of METs conducted by the cassava breeding program at Embrapa Mandioca e Fruticultura. It included a total of 22 cassava genotypes, with six genotypes in the validation phase and 16 commercial varieties, which served as control or reference genotypes (Table 1). The trials were conducted across 17 locations over the crop seasons from 2013 to 2021, and each combination of year and location (Year × Location) was considered as an environment (S1 Table). The evaluations took place in the states of Bahia (BA), Minas Gerais (MG), and Mato Grosso do Sul (MS), which are classified as tropical hot and humid regions. In BA and MG, rainfall is concentrated between April and July, with an average annual precipitation above 1100mm in an irregular pattern. In MS, the rainy season extends from October to March, with a prolonged dry period of 5–7 months. Additional details regarding soil type, geographical coordinates, and climatic variables during the period of conducting the trial, days between planting and harvest, are presented in Table 2.

**Table 1:** Agronomic description of the 22 cassava genotypes evaluated in several locations over the crop seasons from 2013 to 2021.

Genotypes	*Origin	Type (HCN)	Peel color	Pulp color	Inter rind color	Consumption
**New clones**						
BR11-24-156	CNPMF	Bitter	Dark brown	White	Cream	Processing
BR11-34-41	CNPMF	Bitter	Light brown	White	Cream	Processing
BR11-34-45	CNPMF	Bitter	Light brown	White	Cream	Processing
BR11-34-64	CNPMF	Bitter	Dark brown	White	Cream	Processing
BR11-34-69	CNPMF	Bitter	Light brown	White	Cream	Processing
BR12-107-002	CNPMF	Bitter	Light brown	White	Cream	Processing
**Checks**						
BRS Caipira	CNPMF	Bitter	Dark brown	White	White	Processing
BRS Dourada	CNPMF	Sweet	Dark brown	Creme	Light pink	Fresh consunptiom
BRS Formosa	CNPMF	Bitter	Dark brown	White	White	Processing
BRS Gema de Ovo	CNPMF	Sweet	Dark brown	Cream	White	Fresh consunptiom
BRS Kiriris	CNPMF	Bitter	Dark brown	White	White	Dual purpose
BRS Mulatinha	CNPMF	Bitter	Dark brown	White	White	Processing
BRS Novo Horizonte	CNPMF	Bitter	White	White	White	Processing
BRS Poti Branca	CNPMF	Bitter	White	White	White	Processing
BRS Tapioqueira	CNPMF	Bitter	Dark brown	White	Purple	Processing
BRS Verdinha	CNPMF	Bitter	White	White	White	Processing
Cigana Preta	DP	Bitter	Dark brown	White	White	Processing
Correntão	DP	Bitter	Dark brown	White	White	Processing
Corrente	DP	Bitter	Dark brown	White	White	Processing
Eucalipto	DP	Sweet	Dark brown	Cream	White	Fresh consunptiom
IAC-90	IAC	Bitter	White	White	White	Processing
Vassoura Preta	DP	Bitter	Dark brown	White	Cream	Processing

*CNPMF, Embrapa Mandioca e Fruticultura; IAC, Campinas Agronomic Institute; DP, Public Domain.

**Table 2:** Location and description of the cassava genotype evaluation environments from 2013 to 2021.

Environments	Year	Tmax	Tmin	Tav	Annual precipitation	Rh%	W/speed	Sol/rad	Altitude	Latitude	Longitude	soil type
Laje–BA (PP)	2013	29.96	22.32	25.46	1300.09	75.55	1.89	17.09	190	13°09′52”	39°25′59”	LYRd
Laje–BA (NH)	2015	30.46	22.40	25.78	980.08	73.87	1.84	17.83	190	13°09′52”	39°25′59”	LYRd
Laje–BA (SJ)	2016	30.94	22.54	26.02	924.66	72.11	1.89	18.51	190	13°08′36”	39°25′46”	LYRd
Laje–BA (SV)	2017	30.07	22.12	25.39	948.66	72.87	1.99	18.67	190	13°08′36″	39°25′46”	LYRd
Laje–BA (RA1)	2018	30.72	22.36	25.82	835.62	71.91	1.87	17.98	190	13°08′47”	39°17′58”	LYRd
Laje–BA (RA2)	2019	31.16	22.76	26.25	834.08	71.56	1.89	19.38	190	13°09′52”	39°25′59”	LYRd
Laje–BA (Capela)	2020	30.46	22.69	25.89	1022.68	75.02	1.87	17.62	190	13°39′52”	39°25′59”	LYRd
Laje–BA (NH)	2021	29.56	23.54	26.02	908.40	76.23	2.18	17.17	190	13°09′52”	39°25′59”	LYRd
Cruz das Almas–BA	2014	32.09	20.05	25.06	736.79	68.81	2.78	18.73	220	12°39′11”	39°07′19”	LYRd
Cruz das Almas–BA	2018	32.58	20.24	25.39	684.71	67.29	2.72	18.38	220	12°39′11”	39°07′19”	LYRd
Cruz das Almas–BA	2019	32.92	20.54	25.73	801.18	67.78	2.71	18.98	220	12°39′11”	39°07′19”	LYRd
Cruz das Almas–BA	2020	31.70	20.52	25.18	1019.36	73.03	2.61	17.32	220	12°39′11”	39°07′19”	LYRd
Cruz das Almas–BA	2021	31.85	20.28	25.07	857.04	74.61	2.29	15.82	220	12°39′11”	39°07′19”	LYRd
Santo Amaro–BA	2016	30.32	23.22	26.06	779.09	73.23	2.38	19.12	42	12°32′48”	38°42′43”	V
Santo Amaro–BA	2017	29.43	22.81	25.42	1117.68	75.28	2.43	19.26	42	12°32′48”	38°42′43”	V
Valença–BA (NR)	2014	28.64	19.68	23.24	2130.00	74.89	1.92	18.17	39	13°22′26”	39°04′30”	LaD
Valença–BA (NR)	2015	29.22	19.90	23.86	1622.00	73.86	1.84	17.85	39	13°22′26”	39°04′30”	LaD
Valença–BA (NR)	2019	29.55	20.36	24.24	1512.00	71.56	1.89	19.38	39	13°22′26”	39°04′30”	LaD
Governador Mangabeira –BA	2020	25.18	20.52	22.85	1019.36	73.03	2.61	17.32	200	12°34′23”	38°42′53”	LYRd
Florestal–MG	2020	28.64	17.30	22.97	1849.80	74.44	2.42	20.60	815	19°53’12”	44°25’56”	AYRD
Alcobaça–BA	2021	25.96	25.05	25.47	726.34	78.84	4.63	16.95	16	17°31’21”	39°11’53”	PDQS
Alagoinhas–BA	2021	31.86	20.27	25.07	857.04	74.57	2.29	18.93	230	12°07’13”	38°24’35”	PYR
Entre Rios–BA	2021	31.18	21.00	25.04	1104.77	77.78	1.10	16.66	162	11°56’31”	38°05’04”	PYR
Dourados–MS	2021	32.43	17.66	24.05	707.10	64.85	0.24	18.17	469	22°11’16”	54°54’20”	LRDF
Itamarajú –BA	2021	30.25	20.58	24.47	1136.0	77.69	1.00	18.13	112	17°02’21”	39°31’52”	LYRd

Soil class: LYRd—dystrophic red yellow latosol; LaD—dystrophic latosol and dystrophic red yellow podzolic; V—vertisol and red yellow podzolic; PYR—red yellow podzolic and red yellow latosol; PDQS–podzol and dystrophic quartz sands; AYRD–dystrophic red yellow argisol; LRDF—red latosol and dystroferric red latosol.

The soil preparation followed the conventional practices, which involved one plowing, two harrowing, and the creation of planting furrows approximately 20 cm deep. The planting itself was carried out manually, placing the stems horizontally in the planting rows. We used standard cassava stems measuring 16–18 cm, from 10-12-month-old stems that were free of pests and diseases. Fertilization and other cultural practices were performed according to the recommended guidelines for cassava cultivation for the target regions based on soil analysis [21]. The trials were arranged in a randomized complete block design with three replications per trial. Each plot consisted of 4–6 rows with 20–25 plants per row, spaced 0.90 m apart between rows and 0.80 m between plants. All field trials were conducted during the rainy season under rainfed conditions (without supplemental irrigation). The genotypes evaluated are part of the UYT trials from the Embrapa breeding program, selected to meet the target product profile for the development of industrial cassava (starch and cassava flour) adapted to the Northeast and Mid-South regions of Brazil, in rainfed, low to mid-altitude, dry/wet agro-ecologies.

At 12 months after planting, the following traits were evaluated: 1) Fresh root yield (FRY), which is the total weight of all roots in the useful plot (16 plants per plot), measured using a suspended digital scale and adjusted to tons per hectare (t ha^-1^); 2) Shoot yield (ShY), representing the above-ground biomass of all plants in the plot after detaching the roots, including stems, leaves, and petioles, adjusted to t ha^-1^; 3) Dry matter content in the roots (DMC), expressed as a percentage (%), determined using the the gravimetric hydrostatic balance method. A 5 kg sample of roots was cleaned to remove excess soil, then weighed on a digital scale to obtain the weight in air, then the sample was placed in a pre-weighed basket, immersed in water using a hydrostatic balance, and the weight in water was recorded. The following formula was used: $DMC=(158.3\;x\frac{weight\;in\;air}{weight\;in\;air-weight\;in\;water})-142.0$, described by [22]; and 4) Dry root yield (DRY), estimated per plot and measured in t ha^-1^, obtained of the product between FRY and DMC.

### 2.2. Individual analysis of phenotypic data

In the initial step of the analysis, each individual trial was evaluated to assess the quality of the data in terms of accuracy, experimental precision, coefficients of variation, and heritability in the broad sense. This was accomplished using a linear mixed model represented by the equation: $y={\mu 1}_{n}+Xb+Z_{1}r+Z_{2}g+\varepsilon_{jk}$, where *y* is an vector of phenotypic values, including *n* observations, *k* trials, and *j* replicates, with *n* number of plots in each trial; μ is the overall mean; *b* is a vector of fixed effects of trials; *r* is a vector of the random effects of replicates (or blocks) within trials, with *r* ∼ *N*(0, $\sigma_{r}^{2}I_{jk}$), where *N* is a multivariate normal distribution and $\sigma_{r}^{2}$ is the replication variance; *g* is an vector of random effects for the i-th genotype, with *g* ∼ *N* (0, $\sigma_{g}^{2}I_{m}$), where $\sigma_{g}^{2}$ is the total genetic variance; *ε*_*jk*_ is an vector of the random effects of the residuals, with εjk ∼ $N(0,\sigma_{e}^{2}I_{n})$, where $\sigma_{e}^{2}$ is the residual variance. *X*, *Z*_1_ and *Z*_2_ are the incidence matrices for their respective effects, with dimensions *n x k*, *n x jk* and *n x m* for trials, repetitions, and genotypes, respectively; 1_*n*_ is an vector of ones; and *I*_*jk*_, I_*m*_, *I*_*n*_ are identity matrices of their corresponding orders.

Diagnostic plots were generated for each individual trial to identify outliers and assess the homogeneity of the residuals. This step aimed to ensure the quality and suitability of the data for subsequent joint analysis of the trials. Genetic parameters were estimated, and the balance between trials was checked. The number of genotypes evaluated in each year of the VCU trials varied from 19 in 2013 to 68 in 2016, with an average of 22 agronomically important genotypes retained for further analysis. Additionally, between five and eight trials were conducted each year, culminating in a total of 69 trials. For the combined analysis, the trials with the fewest lost plots for the assessed traits were selected. As a result, the number of selected environments varied, with 57, 56, 53, and 59 environments chosen for the characteristics FRY, ShY, DRY, and DMC, respectively. Multiple tools were employed to determine the suitable environments for the joint analysis.

The broad-sense heritability ($\hat{H}²$) was calculated using the formula $\hat{H}²=1-[P\bar{E}V/(2\times \sigma_{g}^{2})]$, as proposed by [23], where $P\bar{E}V$ is the mean variance of the prediction error. The experimental accuracy (Ac) was calculated as $\mathrm{Ac}=\sqrt{1-(P\bar{E}V/\sigma_{g}^{2})}$ following [24]. The coefficient of variation (CV%) was also determined using the formula $CV\%=(\sigma_{e}^{2}/\mu )\;x\;100$, where $\sigma_{e}^{2}$ is the estimate of the residual standard deviation, and *μ* is the overall mean of each trial.

### 2.3. Joint analysis of phenotypic data

For the joint analysis, the adjusted means of the individual tests were used. The model used for the joint analysis was: $y={\mu 1}_{n}+X_{1}s+X_{2}b.s+Z_{1}r.s+Z_{2}g.s+\varepsilon jk$, where *y* is an *n x* 1 vector of phenotypic values em *s* ensaio, onde *n =* $\sum_{i=1}^{s}n_{i}$, em que *n*_*i*_ é o número de parcelas no ensaio *s*; *μ* is the overall mean; *S* is an *S x* 1 vector of the fixed effects of trials; *b*. *s* is a *ks x* 1, vector of the fixed effects of sets within trials; *r*. *s* is a *jks x* 1 vector of the random effects of replicates within sets within trials, with *r*. *s* ∼ $N(0,{⨂}_{i=1}^{s}D_{{r.s}_{i}}⨂I_{jki})$; *g*. *s* is an *ms x* 1 vector of the random effects of genotypes within trials, with distribution *g*. *s* ∼ $N(0,G⨂I_{m})$; εjk is an *n x* 1 vector of residuals, with εjk ∼ $N(0,{⨂}_{i=1}^{s}D_{e_{i}}⨂I_{n_{i}})$; *G* is a VCOV matrix for the effect of genotypes across trials with dimension *s*×*s* [25]. The modeling of matrices *G*, *D*_*r*,*s*_ and *D*_*e*_ was based on an analytic factor structure (*FA*_*k*_), where *k* represents the number of multiplicative components of the model. The matrices *G*, *D*_*r*,*s*_ and *D*_*e*_ are *s*×*s* diagonal VCOV matrices, in which each trial has a specific and independent variance component for the effects of replicates within sets and for the residuals, respectively. *X*_1_, *X*_2_, *Z*_1_ *and Z*_2_ represent incidence matrices, with dimensions, *n x s*, *n x ks*, n *x jks* and n *x ms*, respectively, for their respective effects, and 1_*n*_ is an *n x* 1 vector of 1s (ones), and *I*_*m*_, *I*_*jkl*_, and *l*_*ni*_ are identity matrices with their corresponding orders.

To calculate the overall percentage of genetic variation ($\bar{\nu }$) explained by the analytic factor (*FA*_*k*_), the following model was used: $\bar{\nu }=100\;X\;tr({\Lambda \Lambda }^{T})/({\Lambda \Lambda }^{T}+\psi )$, where *Λ* is the matrix of factor loadings; *λ*_l*k*_ is the k factor loading (*k* = 1,2….*k*) for environment 1; ψ is a diagonal matrix with specific variances for each environment, and *tr* represents the trace of the matrix. The most parsimonious model was selected based on the AIC information criterion, comparing models from the first order (*FA*_1_) to the sixth order (*FA*_6_).

To obtain the VCOV matrix of the effect of genotypes within environments, the *FA*_*k*_ model was used, given by: *G* = (*ΛΛ*^*T*^+*ψ*)⊗***I***_***I***_, where *Λ* is the Ɩ × *k* matrix of fator loading {***λ***_***Ik***_}, where ***λ***_***Ik***_ is the *k*^*th*^ factor loading (*k* = 1,2….*k*) for environment Ɩ; *ψ* is the Ɩ × Ɩ diagonal matrix with specific variances for each; *I*_*I*_ is an identity matrix. *ΛΛ*^*T*^ represents the common variance shared across environments; *ψ* accounts for the unique variances specific to each environment, and ⊗ is the Kronecker product, which is used to extend the matrix dimensions to align with the structure of the data. By using this model, the combined effects of genotypes and environments can be effectively partitioned and analyzed, providing insights into the specific and shared variances that contribute to genotype performance across different environments. This enhances the precision of genotype evaluation and selection in breeding programs.

To assess the genetic correlation between environments ($\rho_{\overset{´}{ƖƖ}}$), the model (*FA*) was used with the terms of the above *G* matrix as follows: $\rho_{\overset{´}{ƖƖ}}={COV}_{\overset{´}{ƖƖ}}/\sqrt{\sigma_{ƖƖ}^{2}}x\sigma_{\overset{´}{ƖƖ}}^{2}$, where ${COV}_{\overset{´}{ƖƖ}}$ is the genetic covariance between trials Ɩ and $\overset{´}{Ɩ}$; and $\sigma_{ƖƖ}^{2}$ and $\sigma_{\overset{´}{ƖƖ}}^{2}$ are the genetic variances within environments Ɩ and $\overset{´}{Ɩ}$ trials, respectively. The factor loadings for the $\tilde{f}$ genotypes and the factor loadings for the $\tilde{\delta }$ environments were calculated based on the work of [9].

### 2.4. Yield stability in MET trials

Latent regression graphs were constructed for eight genotypes, including four clones in the final stage of validation, two local varieties, and two checks. These graphs were used to assess the adaptability and stability of the genotypes across different environments, and the regressed factor loadings were employed [16,26]. Predicted breeding values, representing additive random genetic effects, were added to the average BLUPs of the respective genotypes.

The predicted genetic values reflect the performance of the genotype at a factorial load of a given environment [12]. In order to maximize the proportion of genetic covariance and understand the biological significance of the factor loadings of the environments, principal component analysis (PCA) was conducted. The factors were rotated using the varimax technique [27] to obtain rotated factor loading estimates. The first factor accounted for most of the proportion of genetic covariance between environments, while the second factor was orthogonal (no correlation) to the first and explained the next greatest genetic variation between environments. This process was continued for subsequent factors [28].

### 2.5. Variance component estimates

Additionally, variance component estimates for the joint trials were obtained using the given equation: $\sigma_{p}^{2}=(\sigma_{g}^{2})+(\frac{\sigma_{gxe}^{2}}{E})+(\frac{\sigma_{e}^{2}}{ER})$, where $\sigma_{g}^{2}$ is the genotypic variance; $\sigma_{p}^{2}$ is the phenotypic variance; $\sigma_{gxe}^{2}$ is the genotype × environment interaction variance; $\sigma_{e}^{2}$ is the residual error variance; *E* is the number of environments; *R* is the number of replications. Additional metrics were calculated as follows: 1) genotypic coefficient of variation: CVG%=(σg2X)x100, where *X* is the overall mean; 2) coefficient of determination for G×E interaction effects: $r_{i}^{2}=\sigma_{gxe}^{2}/(\sigma_{g}^{2}+\sigma_{gxe}^{2}+\sigma_{e}^{2})$; 3) residual coefficient of variation: CVr%=(σe2X)x100, 4) genotype-environment correlation: $rge=\sigma_{g}^{2}/(\sigma_{g}^{2}+\sigma_{gxe}^{2})$; 4) CVratio = *CVG*/CVr; and 5) the difference between the phenotypic coefficient of variation (CVP) and the genotypic coefficient of variation (CVG): *P*−*G*. These analyses were performed using the gamem_met() function from the metan package [29].

### 2.6. Correlations between environmental covariates and FA model factor loadings

The correlations between environmental covariates and factor loadings from the FA model provide insights into how specific environmental factors influence the performance of genotypes. These correlations are essential for understanding the underlying mechanisms driving G×E interactions and can guide the selection of genotypes better adapted to specific environmental conditions.

Pearson’s correlation was performed to analyze the relationship between the main environmental covariates influencing the production cycle of cassava, such as maximum temperature (Tmax, °C), minimum temperature (Tmin, °C), average temperature (Tav, °C), rainfall (Rain, mm/day), relative humidity (Rh, %), wind speed (W/speed, m/s), solar radiation (Sol/rad, MJ/m^2^/day), and altitude (m). These covariates were obtained from the meteorological station of Embrapa Mandioca e Fruticultura and the automatic station of Inmet (National Institute of Meteorology). The correlation analysis was performed using the four factors (*FA*_4_) obtained from the structure of the analytical model, following the approach proposed by [30] and described in [12]. Statistical procedures were carried out using the ASReml-R v.3 package [31] within the R4.2.0 software [32].

### 2.7. Comparative approach of AMMI and GGEbiplot with the FA model

The Additive Main Effects and Multiplicative Interaction (AMMI) analysis and the genotype main effects plus genotype × environment interaction effects (GGEbiplot) were conducted as comparative methods to the FA model. The AMMI model [33] used in the analysis is expressed as follows: $y_{ij}=\mu +\beta_{j}+\sum \lambda_{in}\xi_{in}\eta_{in}\epsilon_{ij}$, where, *y*_*ij*_ represents the mean of genotype *i* in environment *j*; μ is the overall mean; *β*_*j*_ is the main effect of environment *j*; *n* is the singular value; *λ*_*in*_, *ξ*_*in*_, and *η*_*in*_ are the singular vectors for genotype and environment for n = 1,2,…n = 1, 2,… respectively; and *ϵ*_*ij*_ is the residual effect. GGE biplots were generated using the first two symmetrically scaled Principal Components (PC) to create average tester coordinate and polygon view biplots. For the GGE model, the equation used is: $y_{ij}=\mu +\alpha_{i}+\beta_{j}+{ɸ}_{ij}$, where *y*_*ij*_ is the mean of genotype *i* in environment *j*, where i = 1…g; j = 1…e, where *g* and *e* are the numbers of genotypes and environments, respectively; μ is the overall mean; *α*_*i*_ is the main effect of genotype *i; β*_*j*_ is the main effect of environment *j*; ɸ_*ij*_ is the interaction effect between genotype *i* and environment *j* [34].

## 3. Results

### 3.1. Individual and joint analysis of the agronomic trials

S1 Fig presents the estimated genetic parameters for individual trials, showing considerable variation across the evaluated agronomic traits. The genetic variance ($\sigma_{g}^{2}$) was found to be high for all traits in all trials. The broad-sense heritability ($\hat{H}²$) ranged from 0.16 to 0.92 for FRY, 0.15 to 0.93 for ShY, 0.28 to 0.92 for DRY, and 0.01 to 0.96 for DMC. In 70% of the trials, the heritability ($\hat{H}²$) surpassed 0.60 for FRY, 0.61 for ShY, 0.61 for DRY, and 0.63 for DMC—values considered notably high for quantitative traits in cassava clones, according to [35].

Regarding experimental precision, the coefficient of variation (CV%) varied from low (<10) to high (>30), for different traits. For FRY, the CV% ranged from 6.60% in 2017.ERU.NH trial to 39.01% in 2020.ERU.NH2B trial. Similarly, for ShY, the CV% varied from 7.84% in 2017.ERU.SA trial to 40.25% in 2013.EC.NH trial. For DRY, the CV% ranged from 6.92% in 2017.ERU.SV trial to 41.78% in 2020.ERU.NH2B trial. In contrast, there was less variation in CV% for DMC, ranging from 1.34% in 2016.ERU.SA trial to 8.41% in 2020.ERU.UFV trial, showing high precision. Notably, the 2015.EC.NR trials for FRY, ShY, and DRY (with CV% of 26.12%, 34.25%, and 28.32%, respectively), demonstrated low experimental precision. As a consequence, this might lead to reduced estimates of $H_{c}^{2}$ (less than 0.31 in the mentioned examples).

Based on the correlation analysis of various experimental precision parameters, CV% and $\hat{H}²$ were the most correlated (r ≥ -0.70) for the four traits (S2 Fig). Correlations for the other parameters were not significant, except for the correlation between $\hat{H}²$ and experimental accuracy for the DMC trait.

In clonal trials, the average heritability estimates were lower for FRY ($\hat{H}²$ = 0.50), DRY ($\hat{H}²$ = 0.51), and ShY ($\hat{H}²$ = 0.54). However, for the DMC variable, the preliminary trial (2020.EP.GS.RA1) showed the lowest $\hat{H}²$ estimate (0.01), while the advanced and uniform trials, i.e. pre-launch phase, generally exhibited $\hat{H}²$ values of 0.70 or higher.

### 3.2. Interpretation of the factor analytic model

In order to efficiently select genotypes, it’s important to estimate variance components that account for the genetic and environmental variations in trait expression. Consequently, the cassava trials were carried out in environments with distinct soil and climatic conditions, which could result in diverse responses among genotypes, aiming to explore the concept of specific adaptability and yield stability of genotypes for a particular target region (Table 2). By joint variance analysis, the results showed high environmental variance for ShY and greater genetic variation for DRY. Additionally, significant G×E interactions were observed for all traits (S3 Fig). The effect of genotypes, environments, and the G×E was highly significant (p<0.001) for all traits based on the maximum likelihood ratio (LTR) test. This significance indicates that the responses of these factors are not uniform, and the average performance of the genotypes varies across different environments. As a result, the ranking of the genotypes changed depending on the specific environmental conditions, emphasizing the importance of carefully analyzing and decomposing the G×E interaction. Failing to account for these interactions could introduce bias in selecting superior genotypes and predicting genetic gains (S2 Table). Therefore, a deeper understanding of G×E is essential to improve selection strategies and recommend genotypes that are both adaptable and stable across target environments.

In order to grasp the intricacies of G×E interactions, we employed the FA model framework. This model effectively breaks down the G×E variation into latent factors representing both genotype and environmental variability. These factors are empirically dependent on the richness of available diversity data, as highlighted in S4 Fig.

The criteria used for selecting the appropriate number of analytic factors in the variance and covariance models indicated that the *FA*_4_ model had the lowest AIC (Akaike Information Criterion) for all analyzed traits. This model was considered the most parsimonious, providing the best fit with the lowest number of estimated parameters (variance-covariance components). The total number of parameters ranged from 233 for DRY to 253 for DMC, taking into account VCOV structures for the estimated G matrix, to elucidate the genetic variation in the dataset (Table 3). Furthermore, the relationship between the last logREML (REML log-likelihood) and the chosen *FA*_4_ model was above 90% for all variables, indicating a good fit and precision.

**Table 3:** Total number of parameters (variance-covariance components—NP), Akaike Information Criterion (AIC) and log-likelihood REML (logREML) of the variance and covariance models (VCOV) based on the estimated G matrix in the joint analysis of environments.

**Fresh root yield**				**Shoot yield**			
Model	NP	AIC	logREML	Model	NP	AIC	logREML
*FA*_1_	114	2969.65	-1370.83	*FA*_1_	112	2897.23	-1336.61
*FA*_2_	160	2973.85	-1326.93	*FA*_2_	155	2847.66	-1268.83
*FA*_3_	201	2939.60	-1268.80	*FA*_3_	200	2813.94	-1206.97
^*^ ***FA***_**4**_	**247**	**2894.00**	-1200.00	***FA***_**4**_	**242**	**2782.27**	**-1149.14**
*FA*_5_	332	2986.08	-1161.04	*FA*_5_	326	2855.00	-1101.50
OneStage	60	12389.10	-6134.54	OneStage	59	11900.12	-5891.06
r*FA*_4*M*_			0.96	r*FA*_4*M*_			0.97
**Dry root yield**				**Dry matter content**			
Model	NP	AIC	logREML	Model	NP	AIC	logREML
*FA*_1_	106	1403.20	-595.60	*FA*_1_	118	1027.04	-395.52
*FA*_2_	147	1385.43	-545.71	*FA*_2_	159	997.40	-339.70
*FA*_3_	192	1367.67	-491.84	*FA*_3_	207	1000.80	-293.40
***FA***_**4**_	**233**	**1361.68**	**-447.84**	***FA***_**4**_	**253**	**985.22**	**-239.61**
*FA*_5_	308	1422.78	-403.39	*FA*_5_	344	1074.14	-193.07
*FA*_6_	356	1393.97	-340.99	OneStage	62	4138.70	-2007.35
r*FA*_4*M*_			0.97	r*FA*_4*M*_			0.91

FA (k: analytic factor for the model of order k; * Chosen model based on the lowest AIC values (in bold).

The first four factors of *FA*_4_, were responsible for R^2^ > 87% of the observed genetic variance, making it possible identify approximately 25 environments with high loadings across the four traits. For example, for FRY, the first factor had loadings ranging from -1.74 (2021.EC.GS.UFRB) to 16.53 (2020.ERU.UFV). Considering loadings over 5.0, twelve environments were represented (including three clonal trials, two preliminary trials, one advanced trial, and six uniform trials). The second factor had two environments with loadings > 5.0 (not present in factor 1), being them 2018.ERU.RA1 and 2021.ERU.ALC. The third factor had five environments, and the fourth factor had six environments, all with negative loadings. Similar trends were observed for ShY, with approximately five environments in each factor and loadings ranging from -1.58 (2021.EP.WX.RA1) to 13.25 (2013.EC.NH).

### 3.3. Variance components

The genetic parameters estimated in the joint trials indicate the extent of genetic progress achieved through the breeding process. Therefore, the values suggested that most traits exhibited higher *CVr* compared to *CVg*, implying that the phenotypic variance is more influenced by unknown sources of variability, which could be micro and macroenvironmental differences, rather than genetic correlations. However, DMC displayed smaller variations and a *CVg*/*CVr* ratio of 1.33, indicating it is more responsive to selection and genetic gain [S3 Table]. The *CVr* values ranged from 23.84% for ShY to 3.14% for DMC, while the *CVg* ranged from 19.14% for FRY to 4.20% for DMC. The broad-sense heritability ($\hat{H}²$ ranged from 0.15 for ShY to 0.31 for DMC, indicating a significant environmental effect on root yield and quality traits.

In an exploratory analysis, it was observed that the average in FRY ranged from 11.71 t ha^-1^ (2016.ERU.SA) to 38.78 t ha^-1^ (2020.ERU.UFV), with an overall average of 24.14 t ha^-1^. The genotype BR11-34-69 had the highest overall average for this trait (33.65 t ha^-1^), which is well above the national average of 15.0 t ha^-1^ [36]. For ShY, the range was from 10.39 t ha^-1^ (2016.ERU.SA) to 45.53 t ha^-1^ (2021.ERU.ITAM), with an overall average of 21.40 t ha^-1^. The genotype BRS Poti Branca had the highest overall average (22.86 t ha^-1^). For DRY, the variation ranged from 3.50 t ha^-1^ (2016.ERU.SA) to 12.56 t ha^-1^ (2021.ERU.NH4), with an overall average of 7.61 t ha^-1^. The genotype BR11-34-41 had the highest overall average (9.36 t ha^-1^). The DMC trait showed the least variation, ranging from 30.79% (2021.ERU.AL) to 38.26% (2019.ERU.NR), with an overall average of 35.63% across the trials. The genotype BRS Novo Horizonte stood out with an overall average of 38.0% (S5 Fig).

### 3.4. Correlations among the agronomic trials

**Fig 1:**
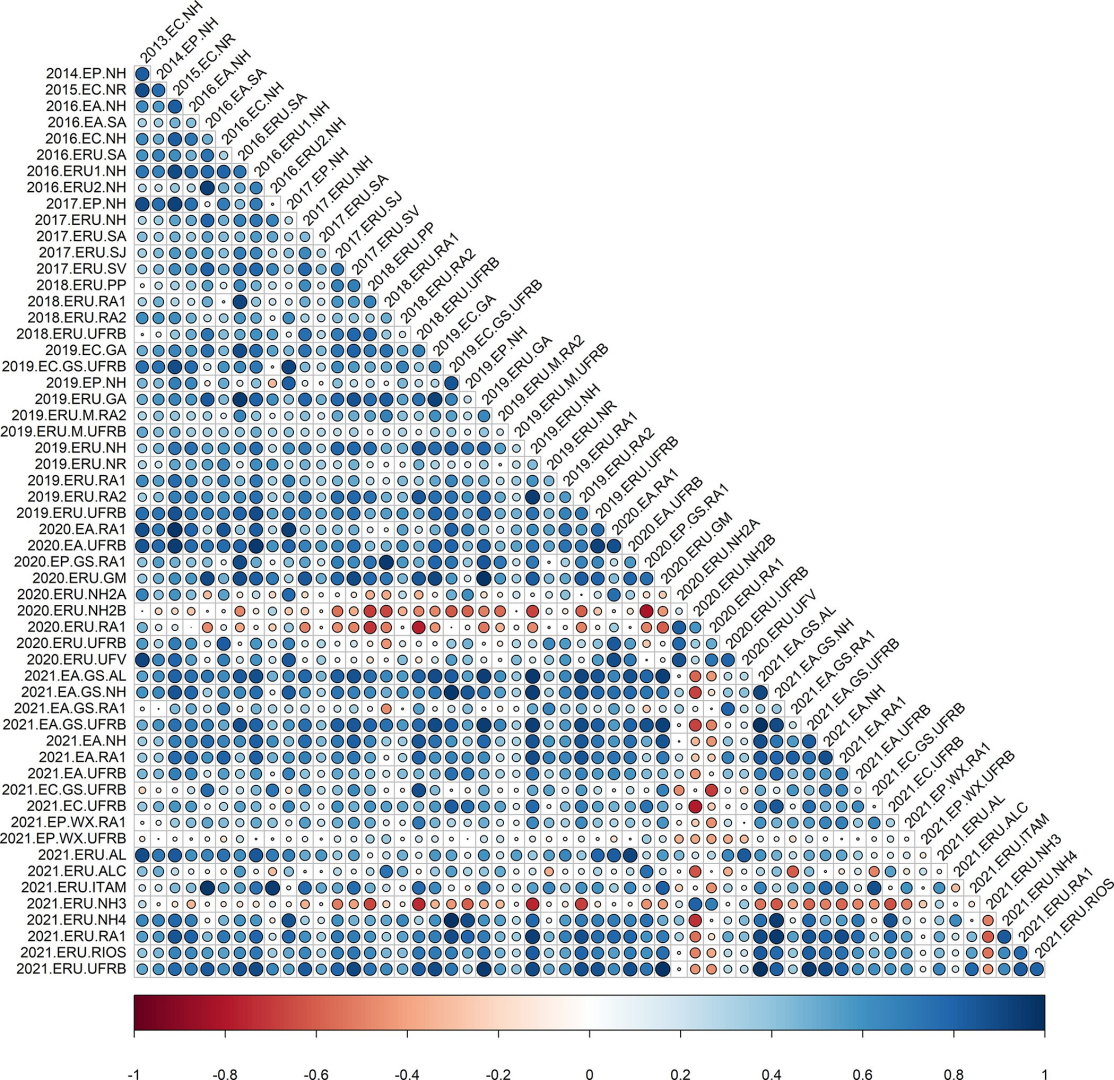
Estimated pairwise genetic correlations between pairs of environments for fresh root yield (FRY) across 57 trials. The circles correspond to the magnitude (size) and direction (color) of genetic correlations between environments, respectively.

**Fig 2:**
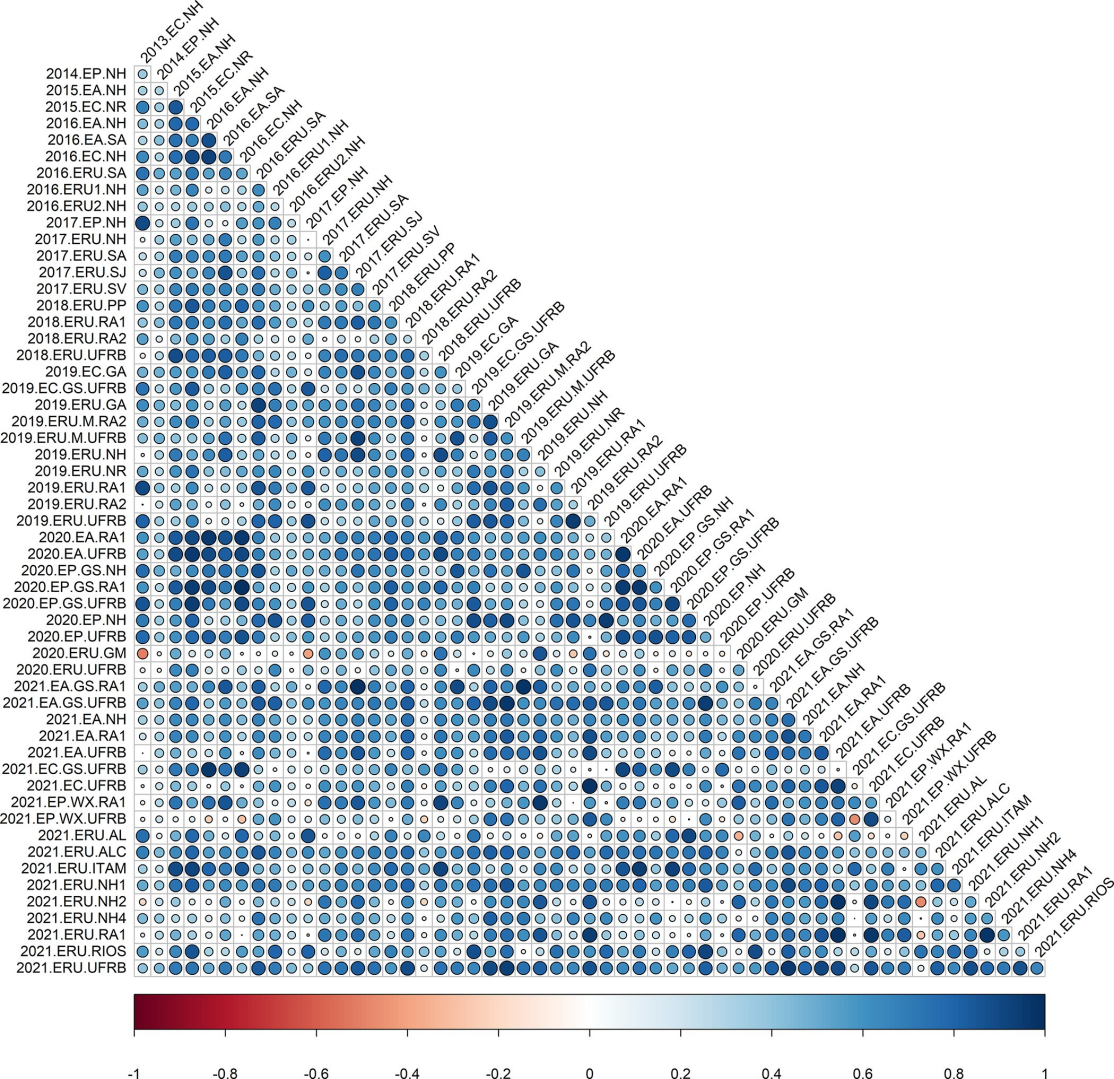
Estimated pairwise genetic correlations between pairs of environments for shoot yield (ShY) across 56 trials. Circles correspond to the magnitude (size) and direction (color) of genetic correlations between environments, respectively.

**Fig 3:**
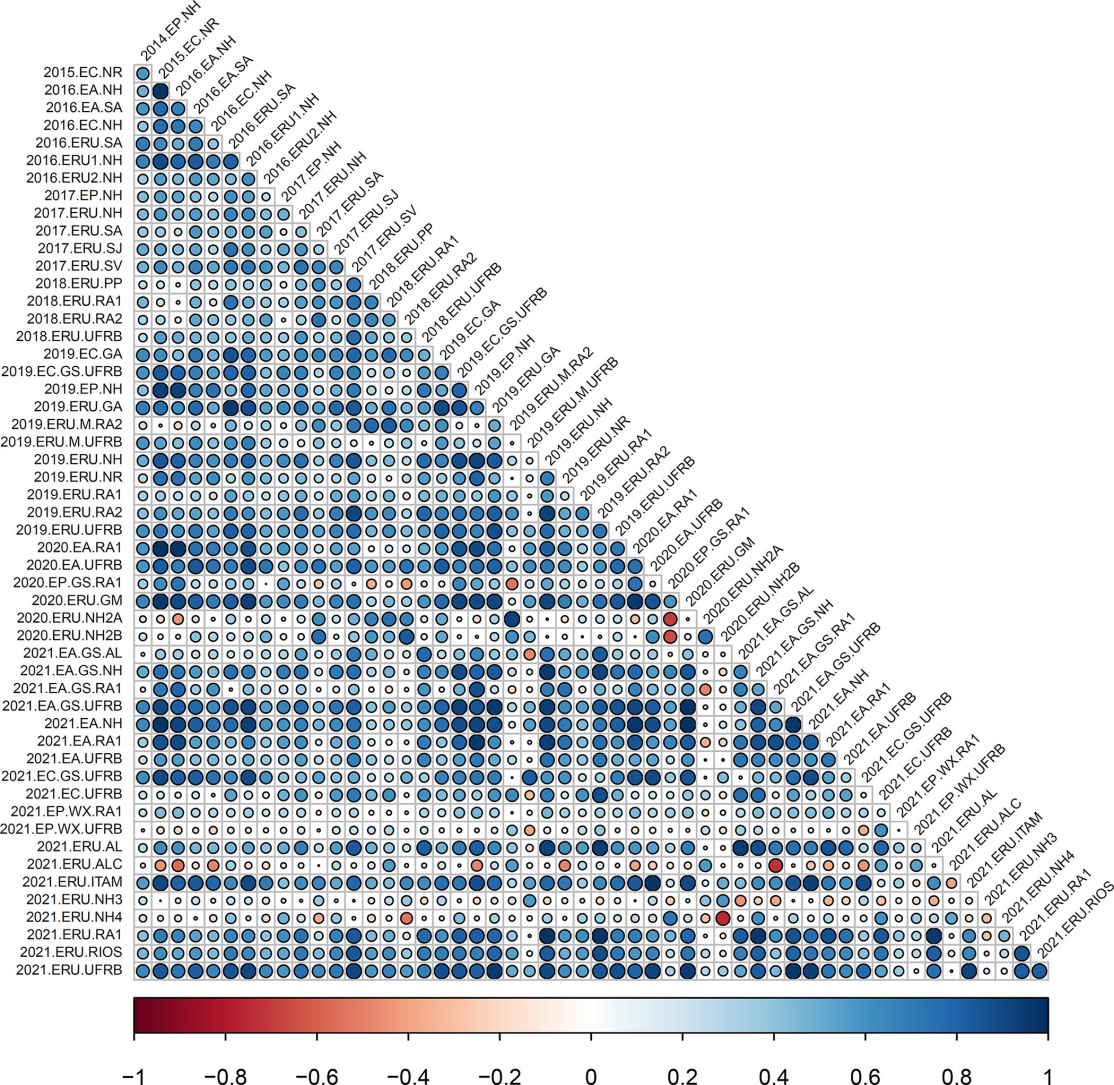
Estimated pairwise genetic correlations between pairs of environments for dry root yield (DRY) across 53 trials. Circles correspond to the magnitude (size) and direction (color) of genetic correlations between environments, respectively.

**Fig 4:**
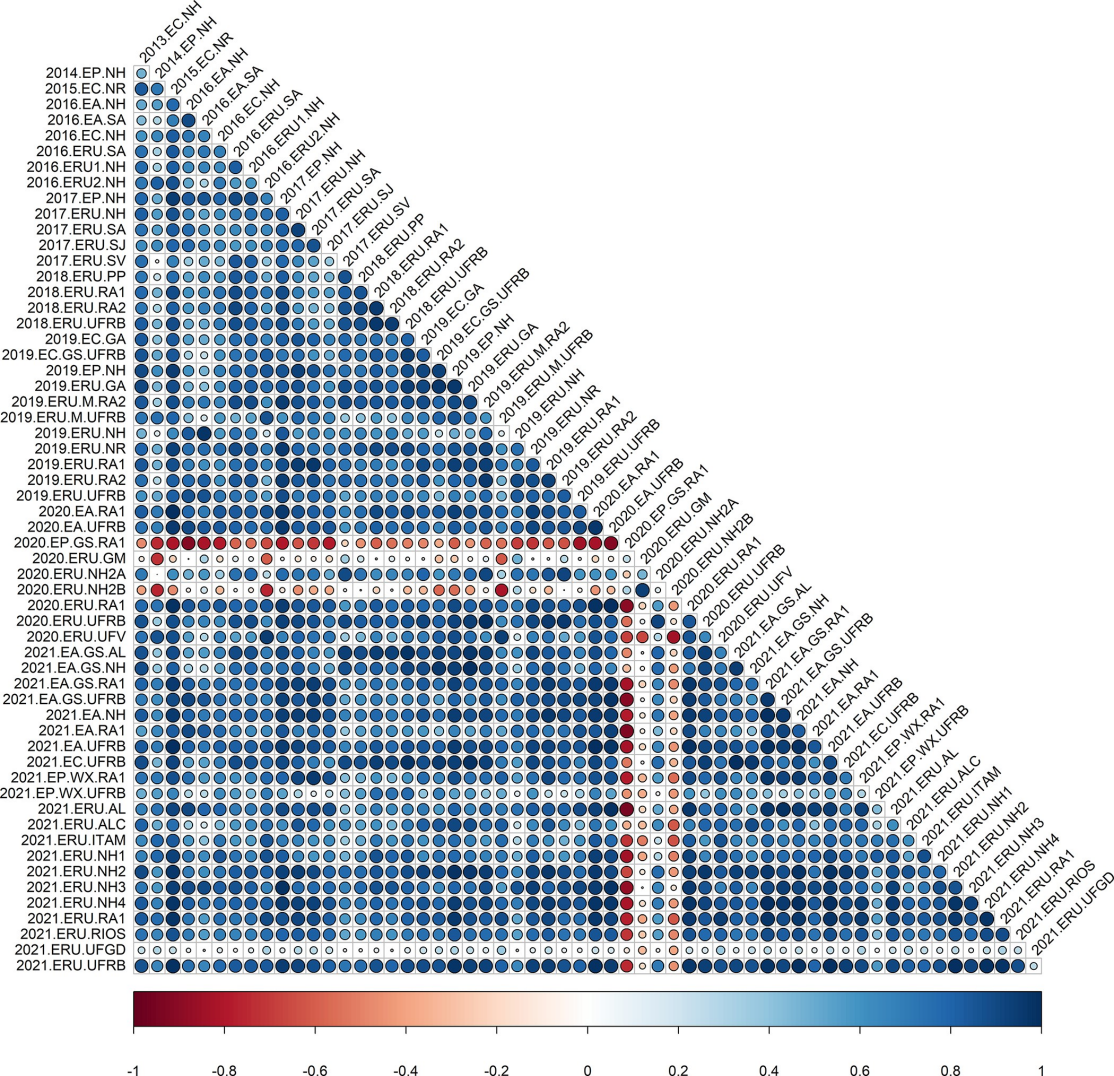
Estimated pairwise genetic correlations between pairs of environments for cassava dry matter content in the roots (DMC) across 59 trials, circles correspond to magnitude (size) and direction (color) of genetic correlations between environments, respectively.

The analysis of correlations using the analytic factor (*FA*_*k*_) demonstrated significant variation in the magnitude and direction of trials associations for all traits (Figs 1–4), as indicated by the decomposition of G×E by the FA model. The correlations also highlighted robust relationships between trials, even when conducted across different years (2013 to 2021), underscoring the necessity of utilizing the FA model for such datasets. For instance, comparing field trials like 2013.EC.NH and 2021.ERU.AL displayed correlations exceeding 90% for FRY and ShY. Similarly, trials such as 2015.EC.NR with 2021.EA.NH exhibited strong correlations for DRY and DMC.

For FRY, the majority of correlations (69.87%) were positive and significant (ranging from 0.25 to 0.99), indicating a low to no difference in genotype performance across environments. Only a small proportion (11.02%) of correlations were negative and significant (ranging from -0.26 to -0.82). Generally, genetic correlations between 0 < r < 0.20 imply a strong crossover G×E, meaning the variance of the interaction is so high that it surpasses all major genetic effects, altering the genotype rankings across environments. This phenomenon was observed in 15.16% of the environmental correlations for FRY, ranging from -0.20 in the environments 2020.ERU.UFV and 2018.ERU.PP to 0.20 between 2021.EA.GS.RA1 and 2017.ERU.SA. Conversely, when genetic correlations fall within the range of 0.20 < r < 0.80, it suggests the presence of non-crossover G×E interactions, signifying that the variance attributed to the G×E interaction is of lesser significance. Correlations of such magnitude were notably observed in trials such as 2016.EC.NH and 2014.EP.NH, where a correlation coefficient of r = 0.50 was observed for the trait FRY.

For ShY, there were fewer pairs of environments with negative genetic correlations compared to FRY. Instead, there was a high concentration of average genetic correlations, indicating that the variance of the G×E interaction is not as significant, thereby altering the quantitative difference among genotypes and some of the rankings among environments. For example, the correlation of r = -0.50 between 2020.ERU.GM and 2013.EC.NH demonstrated a moderate negative relationship between these environments.

Finally, when r > 0.80, noncrossover G×E interactions predominate, indicating minimal variance in the interaction as major genetic effects explain a substantial portion of the genetic variability. Consequently, there is little to no change in genotype classification among environments, given that major genetic effects predominantly contribute to the genetic variability (observed in 34.96% of the evaluated environments). For instance, in the case of FRY, a positive correlation of 0.99 between 2021.EA.GS.UFRB and 2021.EA.GS.AL suggests a high level of genotype stability between these environments.

For DRY, the negative environmental correlations ranged from r = -0.01 (2021.ERU.ALC × 2021.ERU.AL) to -0.76 (2021.ERU.NH4 × 2020.ERU.NH2B), with significance observed in 4.78% of the negative genetic correlations between environments. There was a perfect correlation between 2020.EA.RA1 and 2015.EC.NR, and a correlation of 0.0 between 2020.EP.GS.RA1 and 2018.ERU.RA1. The positive and significant correlations were observed in 71.22% of the correlations analyzed (Fig 3).

Regarding DMC, there was a high concentration of positive and significant genetic correlations between the tested pairs of environments, accounting for 73.29% of the correlations. There was also a significant proportion of high correlations. Only 4.5% of the correlations were below 0.20 (Fig 4). The negative environmental correlations ranged from -0.01 between 2020.ERU.NH2B and 2018.ERU.PP to -0.95 between 2021.ERU.AL and 2020.EP.GS.RA1, with only 2.92% of them being significant. The positive correlations varied from 0.0 between 2020.ERU.NH2A and 2014.EP.NH to 1.0 between 2020.EA.RA1 and 2015.EC.NR, with 85.35% of them being significant.

### 3.5. Stability of cassava genotypes for fresh root yield

The latent regression plots based on the predicted genetic values (Y coordinate) regressed from the environmental loadings (X coordinate) obtained from the *FA*_4_ model were used to assess the magnitude of G×E (or stability) for eight genotypes evaluated in at least 50% of the environments. These genotypes were selected based on their predicted mean agronomic performance and importance for the cassava breeding program. They are BR11-34-41, BR11-34-45, BR11-34-64, BR11-34-69, BRS Novo Horizonte, BRS Poti Branca, Cigana Preta, and Corrente. The analysis focused on four agronomic traits: FRY, ShY, DRY, and DMC. The factors of the *FA*_4_ model collectively explained between 87.31% (DRY) and 95.52% (DMC) of the proportion of the genetic variance of the observed G×E.

The regression slope (*β*_1_) indicates the sensibility and possible causality among the genotype’s sensibility and the factor’s signal. A high and positive slope indicates that the genotype is more responsive to environmental improvements, resulting in higher predicted values in environments with higher factor loadings. This suggests that the genotype is well adapted to such environments, as indicated by a higher angular coefficient [S4 Table]. In our analysis, the estimated environmental loadings for the first factor are mostly positive, with only five exceptions. Therefore, positive slopes for this factor are desirable.

**Fig 5:**
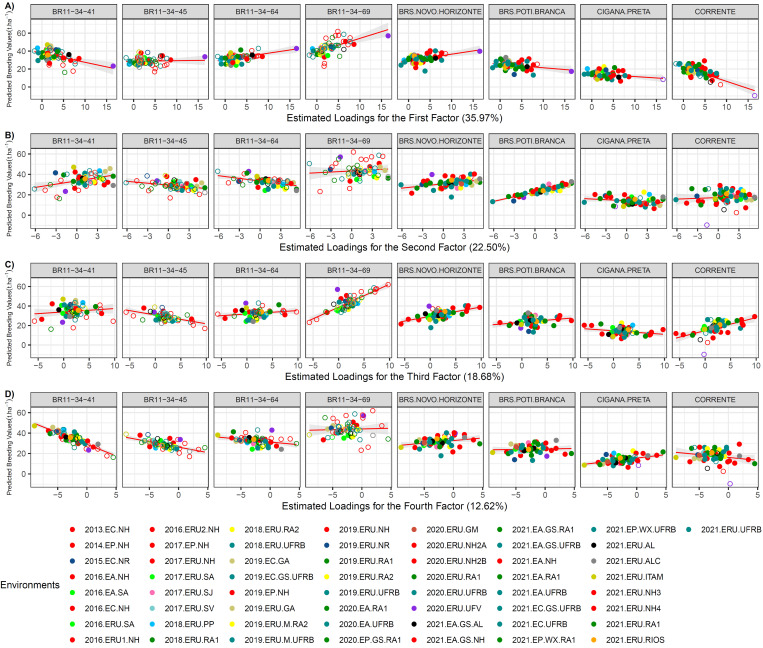
Latent regression plots for fresh root yield (FRY) for: A) first analytic factor; B) second analytic factor; C) third analytic factor and D) fourth analytic factor. The solid and empty circles correspond to the predicted genetic values of the genotypes at tested and untested locations, respectively. The solid red line and gray tone correspond to the latent regression line at the 95% confidence interval, respectively. In parentheses the proportion of the genetic variance explained for each factor.

For FRY, the genotypes BR11-34-69 and BRS Poti Branca showed the highest responsiveness to improved environments, with *β*_1_ values of 1.49 and 1.52, in the first and second FA, respectively [Fig 5]. These genotypes are recommended for environments with the highest factorial loadings, ranging from 5.02 (2020.EA.RA1) to 16.53 (2020.ERU.UFV) for the factor 1, and two environments with 5.28 (2021.ERU.ALC and 2018.ERU.RA1) for the factor 2. The average FRY in these environments was 23.0 t ha^-1^ (S5 Fig). Genotypes with regression line slopes (*β*_1_) close to zero are considered more stable, even in the face of environmental improvements. For the set of environments related to the first factor, the genotypes BR11-34-45, BR11-34-64, and BRS Novo Horizonte, were identified as the most stable for FRY. To the second factor, we can highlight the genotypes BR11-34-69, BRS Novo Horizonte, and Corrente, exhibiting *β*_1_ less than 1 to FRY (with positive loadings).

In the third factor, the genotypes BR11-34-69 and Corrente exhibited higher responsiveness to the corresponding environments, while BR11-34-41 showed responsiveness in the opposite direction for the fourth factor. Conversely, for the set of environments associated with the third factor, the most stable genotypes were BR11-34-41 and Cigana Preta with *β*_1_ values equal to 0.35 and -0.37, respectively. For the fourth factor, BRS Poti Branca with *β*_1_ = 0.09 was identified as the most stable genotype, to these environments. These genotypes can be recommended for environments with specific loadings, such as six environments with loadings ranging from 6.26 (2021.EA.RA1) to 9.71 (2019.EP.NH) in the third factor, exhibiting an average FRY of 27.76 t ha^-1^. Additionally, seven environments in the fourth factor, with values between -5.07 (2016.EA.SA) and -8.9 (2021.ERU.ITAM), were also identified, demonstrating changes in the performance of genotypes between environments, with an average FRY of 25.31 t ha^-1^.

The change in the slope of the regression line and the dispersion of environments along the regression line for the evaluated clones provide insights into the genotype’s response in different environments for FRY (Fig 5). For example, the clone BR11-34-69 exhibited a positive and high magnitude slope, indicating a positive correlation between FRY and FA1 for the environments associated with the first factor, consistent with its higher performance. However, for the set of environments in the fourth factor, BR11-34-69 showed no change in classification due to its high stability. On the other hand, BR11-34-41 exhibited less responsiveness, as indicated by the negative slope for environments in factors 1 and 4, as well as greater dispersion along the regression line for *FA*_3_. However, there was a positive and low magnitude slope for environments in the second and third factors, indicating a positive response and suggesting stable performance of BR11-34-41.

### 3.6. Stability of cassava genotypes for shoot yield

Regarding the trait ShY, the analysis identified two new genotypes, for *FA*_1_, in which BR11-34-41 and BR11-34-69, as the most responsive to environmental improvement. The *FA*_1_ explained 38.05% of the genetic variance. These genotypes exhibited *β*_1_ values of 1.48 and 2.05, respectively. Additionally, BRS Poti Branca showed a high responsiveness to environmental improvement (*β*_1_ = 2.55) for the second factor (*FA*_2_), which accounted for 25.29% of the genetic variance. Collectively, BR11-34-41, BR11-34-64, BR11-34-69, and Cigana Preta proved to be the most responsive and adapted genotypes for the environments influenced by the third factor (17.86%), with *β*_1_ values of 1.91, 1.65, 1.52, and 1.35, respectively. In the case of the fourth factor, Corrente (*β*_1_ = 2.15) and BR11-34-69 (*β*_1_ = -2.85) exhibited hight and contrasting responsiveness. Therefore, the BR11-34-69 genotype could be useful for characterizing environments with extreme positive and negative loadings for the first and fourth factors affecting ShY.

In general, the genotypes BRS Novo Horizonte, BRS Poti Branca, and Cigana Preta demonstrated similar responses to the environmental factors (*β*_1_ ≅ 0). Among them, only BRS Poti Branca displayed consistent behavior for both the first and third factors (*β*_1_ = 0.23 and 0.24, respectively). BRS Novo Horizonte, due to its similar response across a wide range of environments, also exhibited values close to zero (*β*_1_ = 0.26) for the fourth factor.

The first factor represented environments with environmental loads ranging from 5.95 (2021.ERU.RIOS) to 13.25 (2013.EC.NH), while the second factor encompassed loads from 4.99 (2021.ERU.ALC) to 9.33 (2019.ERU.M.UFRB), with shoot yield exceeding 23 t ha^-1^ (S4 Fig). The third factor comprised seven environments with loads between 6.31 (2015.EA.NH) and 10.43 (2016.EC.NH), exhibiting an average Shy of 29.18 t ha^-1^. Similarly, the fourth factor consisted of six environments with loads ranging from -6.43 (2013.EC.NH) to 8.22 (2019.ERU.NH), and an average ShY of 22.80 t ha^-1^.

**Fig 6:**
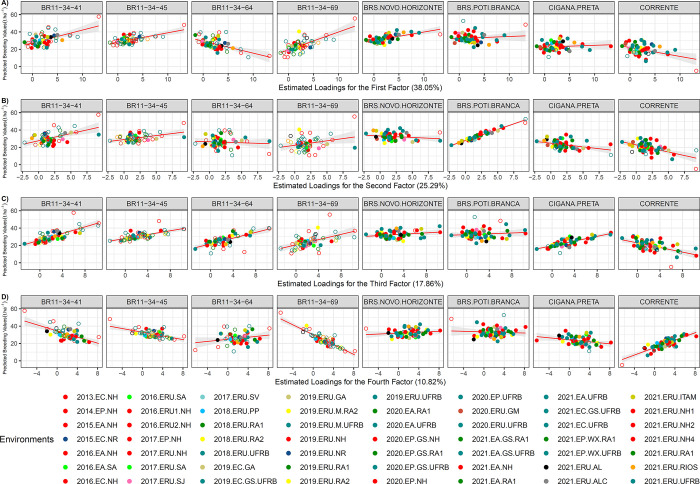
Latent regression plots for shoot yield (ShY) for: A) first analytic factor; B) second analytic factor; C) third analytic factor and D) fourth analytic factor. The solid and empty circles correspond to the predicted genetic values of the genotypes at tested and untested locations, respectively. The solid red line and gray tone correspond to the latent regression line at the 95% confidence interval, respectively. In parentheses the proportion of the genetic variance explained for each factor.

Regarding the regression analysis, the genotypes BR11-34-41, BR11-34-45, and BR11-34-69 displayed positive and upward slopes for the loadings of the first, second, and third factor environments, respectively. Conversely, Corrente variety exhibited a negative slope in the first three factors but displayed favorable performance in the fourth factor. BRS Novo Horizonte displayed a high and positive *β*_1_ for environments grouped in the first factor, indicating its greater responsiveness to these environments. However, the same variety exhibited a low *β*_1_ for environments grouped in the other three factors, indicating high yield stability (with a low magnitude of the regression line) across environments influenced by these three factors (Fig 6).

### 3.7. Stability of cassava genotypes for dry root yield

Based on the *FA*_4_ model, which the first factor explains 38.34% of the genetic variance, the genotypes BR11-34-69 and BR11-34-41 exhibited a high level of responsiveness to DRY (β₁ = 1.61 and 1.30, respectively). Conversely, the genotypes Cigana Preta and Corrente displayed poor agronomic performance under improved environmental conditions (β₁ = -1.32 and -1.92, respectively). Furthermore, BR11-34-45 and Corrente demonstrated significant responsiveness to the second and fourth factors (β₁ = 1.41 and 2.30, respectively). Notably, the genotype BR11-34-41 exhibited β₁ < 1 (but positive) across the second to fourth factors, indicating considerable stability in at least 37.7% of the environments categorized within these factors (with loadings >1.5) (S4 Fig).

BR11-34-69 and BRS Poti Branca exhibited high stability in factors 4 and 1 (β₁ = 0.08 and 0.32, respectively). Conversely, BRS Novo Horizonte displayed greater stability in the first two factors (β₁ = -0.19 and -0.66), while BRS Poti Branca showed stability primarily in the third and fourth factors (β₁ = -0.32 and -0.55).

Among the identified environments with high environmental loadings (>1.5), 25 stood out, particularly 2019.ERU.M.UFRB (2.67) and 2019.EC.GS.UFRB (2.46), indicating relatively consistent edaphoclimatic conditions and a phenotypic mean above 7.0 t ha^-1^ for the same year and location.

**Fig 7:**
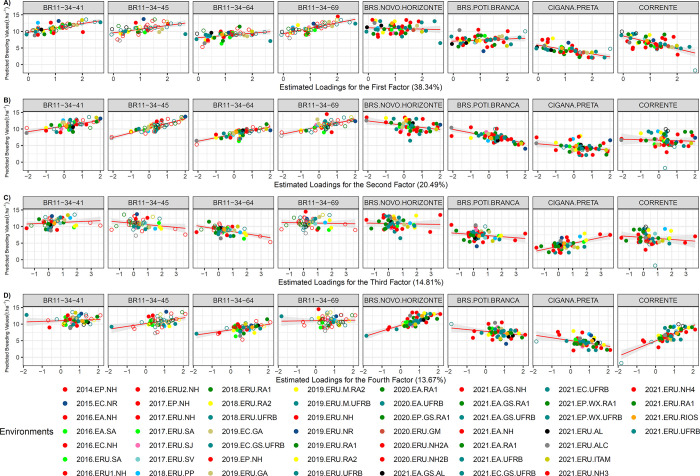
Latent regression plots for dry root yield [DRY] for: A] first analytic factor; B] second analytic factor; C] third analytic factor and D] fourth analytic factor. The solid and empty circles correspond to the predicted genetic values of the genotypes at tested and untested locations, respectively. The solid red line and gray tone correspond to the latent regression line at the 95% confidence interval, respectively. In parentheses the proportion of the genetic variance explained for each factor.

Different cassava genotypes exhibited varied responses in terms of both direction and magnitude of regression. For instance, four new genotypes—BR11-34-41, BR11-34-45, BR11-34-64, and BR11-34-69—showed high responsiveness with a positive slope in environments associated with the first, second, and fourth factors, while the third factor environments displayed low regression line dispersion for these genotypes. Conversely, BRS Poti Branca and Cigana Preta exhibited a negative slope in three out of the four factors, while BRS Novo Horizonte demonstrated a high slope in fourth-factor environments and displayed stability in third-factor environments, along with notable dispersion across the first three factors (Fig 7).

### 3.8. Stability of cassava genotypes for dry matter content in roots

The trait DMC exhibited significant variability in responsiveness to the environment, with β₁ ranging from -2.31 to 0.01. Although most genotypes displayed a negative angular coefficient for factors 1 and 2, indicating a reduction in DMC in environments with high positive loadings. However, BRS Novo Horizonte demonstrated responsiveness to environmental improvement in *FA*_1_ and *FA*_3_ environments (*β*_1_ > 1). Moreover, BRS Novo Horizonte maintained a relatively stable ranking across 29 environments of *FA*_1_ with positive loadings, ranging from 1.03 (2021.EP.WX.RA1) to 2.81 (2021.EA.GS.AL). Lower-magnitude loadings suggest less environmental variation for DMC, which is supported by lower heterogeneity of variance among environments, ranging from 7.99 (2021.EA.GS.AL) to 0.01 (2020.EP.GS.RA1), with a phenotypic environmental mean of 35.54% (S4 Fig). In contrast, the environmental variance heterogeneity for FRY ranged from 99.59 (2020.ERU.UFV) to 8.96 (2021.EA.GS.AL).

**Fig 8:**
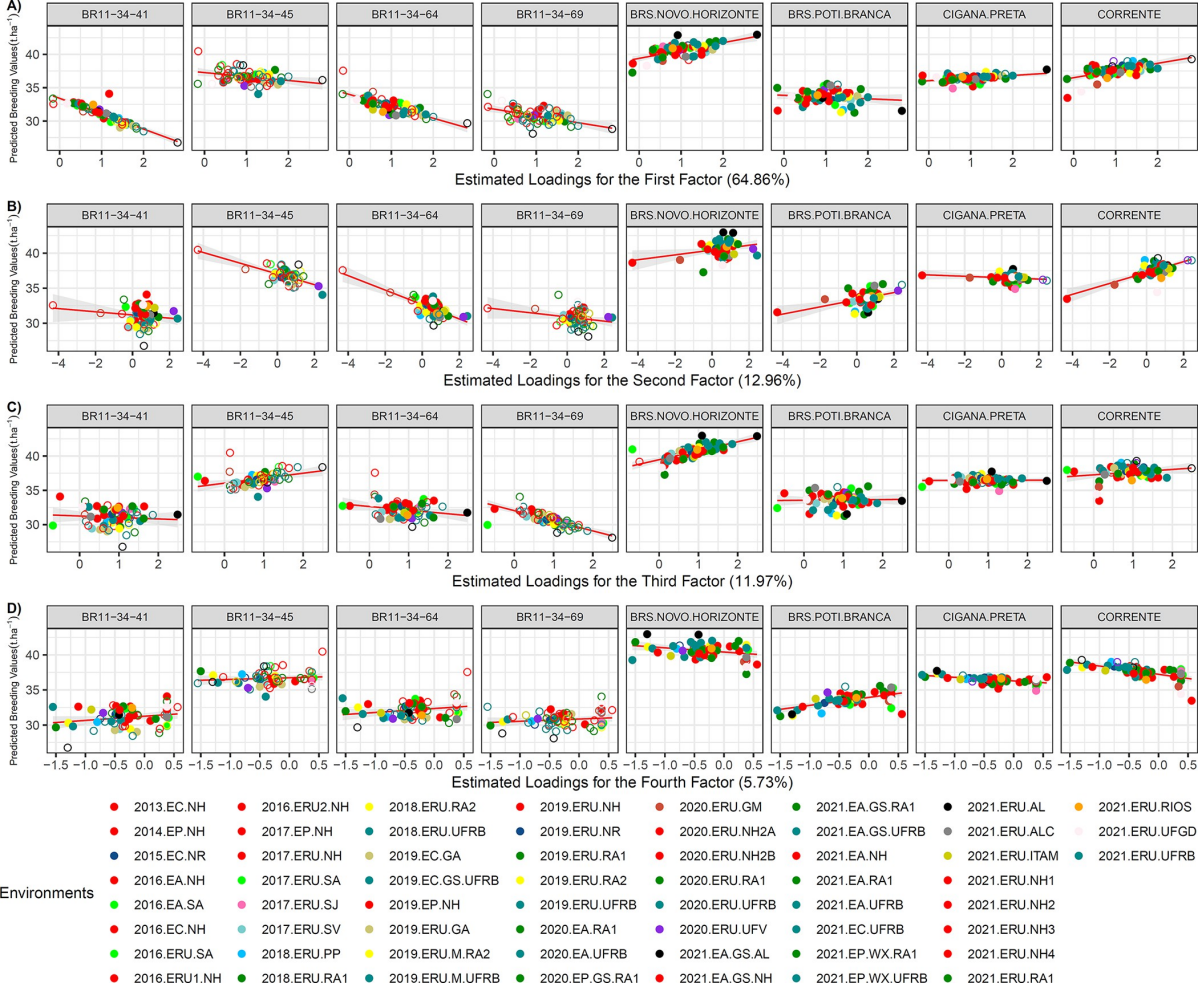
Latent regression plots for dry matter content [DMC] for: A] first analytic factor; B] second analytic factor; C] third analytic factor and D] fourth analytic factor. The solid and empty circles correspond to the predicted genetic values of the genotypes at tested and untested locations, respectively. The solid red line and gray tone correspond to the latent regression line at the 95% confidence interval, respectively. In parentheses the proportion of the genetic variance explained for each factor.

Six out of the eight genotypes exhibited high stability for DMC in at least three out of the four *FA*s, across more than 50% of the evaluated environments. Examples include the genotypes BR11-34-41 and BR11-34-45, as well as the varieties BRS Poti Branca, Corrente, and Cigana Preta (with *β*_1_ ~ 0.43). Generally, there was significant variation in the direction and magnitude of the regression among the genotypes across the evaluated environments. However, BRS Novo Horizonte and Corrente demonstrated higher responsiveness to *FA*_1_ environments, while the others displayed less responsiveness to environmental improvement across most *FA*s (Fig 8).

In terms of biological responses, the biplot analysis revealed several correlated environments for the four agronomic traits. For instance, in relation to FRY, trials originating from NH, UFRB, and UFV environments generally exhibited slightly higher yield, with higher magnitudes of predicted genetic values and higher environmental loadings in FA1 and FA3 (> 50%).

For both DRY and DMC, there was a concentration of high and positive loadings in FA1 and FA2, particularly in environments such as NH, ITAM, NR, and RA1. Additionally, for DMC, environments AL, NH, UFRB, and RA1 displayed positive loadings in *FA*_1_ and *FA*_3_.

Analyzing the dispersion of environments in the regression graph, it was observed that environments RA2 and AL demonstrated low-magnitude dispersion (stability) along the regression line across all four factors for FRY, while for ShY, this behavior was observed in five other environments (GA, GM, NR, PP, SA). For both DRY and DMC, the most stable environments were RA2, AL, ALC, and GM, with a more uniform distribution of predicted genetic values along the regression line. These results reinforce the factor analytical model’s ability to capture the heterogeneity of variance across environments [26].

### 3.9. Correlation between environmental loads and climate variables

Table 4 presents the Pearson’s correlation between climate variables and environmental loads for the *FA*_4_ model, aiming to evaluate the G×E and its influence on genotype performance. The analysis revealed distinct responses of the genotypes for each trait examined. In terms of FRY, the most important environmental covariates explaining the G×E effect and the variation trend were rainfall (Rain, mm day^-1^), ALT (m), and Sol/rad (MJ/m^2^ day^-1^). These variables displayed a positive correlation with environments grouped in the first factor (0.58, 0.55, and 0.32, respectively) and a negative correlation with environments in the second factor (-0.29 for Rain).

**Table 4:** Pearson’s correlation coefficient between the environmental covariates and the environmental loadings of the *FA*_4_ model on 22 cassava genotypes in 55 field trials.

Fresh root yield					Shoot yield				
Variables	*FA*_1_	*FA*_2_	*FA*_3_	*FA*_4_	Variables	*FA*_1_	*FA*_2_	*FA*_3_	*FA*_4_
Maximum temperature	-0.09	-0.05	0.09	-0.10	Maximum temperature	0.17	0.05	0.08	-0.05
Minimum temperature	-0.24	0.16	0.12	0.12	Minimum temperature	-0.18	0.20	-0.14	0.05
Average temperature	-0.23	0.12	0.19	0.08	Average temperature	-0.07	**0.28** ^*^	-0.10	0.02
Rainfall	**0.58** ^***^	**-0.29** ^*^	-0.20	0.15	Rainfall	0.23	0.00	0.18	-0.25
Relative humidity	0.01	-0.12	-0.15	0.18	Relative humidity	-0.05	-0.10	0.01	0.04
Wind speed	0.03	0.19	-0.01	0.22	Wind speed	0.06	0.21	-0.06	0.09
Solar radiation	**0.32** ^*^	0.07	0.01	-0.14	Solar radiation	-0.01	0.08	0.02	-0.10
Altitude	**0.55** ^***^	-0.14	-0.01	0.15	Altitude	0.01	0.05	-0.03	0.00
Dry root yield					Dry matter content				
Variables	*FA*_1_	*FA*_2_	*FA*_3_	*FA*_4_	Variables	*FA*_1_	*FA*_2_	*FA*_3_	*FA*_4_
Maximum temperature	0.12	0.11	0.12	0.04	Maximum temperature	0.23	0.17	**0.26** ^*^	-0.32*
Minimum temperature	-0.14	-0.26	-0.06	0.00	Minimum temperature	-0.10	-0.18	-0.14	0.30*
Average temperature	-0.04	-0.19	0.02	0.03	Average temperature	0.05	-0.06	-0.03	0.10
Rainfall	0.07	**0.28** ^*^	0.04	-0.01	Rainfall	-0.11	0.07	-0.01	0.01
Relative humidity	**-0.31** ^*^	-0.12	-0.13	0.01	Relative humidity	-0.16	-0.02	-0.01	0.13
Wind speed	-0.03	-0.32*	-0.15	-0.13	Wind speed	0.19	0.10	0.01	0.03
Solar radiation	0.26	0.12	0.15	0.09	Solar radiation	0.19	0.12	0.06	0.06
Altitude	0.03	0.03	-0.07	-0.23	Altitude	-0.01	0.21	0.06	-0.13

*; **; *** significant at p<0.05, 0.01 and 0.001% respectively; *in bold are the significant correlations for each environmental covariate for the four analytic factor models; FA = analytic factor for the model of order 4.

For ShY, the only significant correlation was observed with environments in the second factor and Tav (average temperature) with a correlation coefficient of 0.28. Regarding DRY, significant correlations were found for climatic variables Rh (%) with a negative correlation (-0.31) and W/speed (wind speed) with a negative correlation (-0.32) in environments of the first and second factors, respectively. However, for environments in the second factor, a positive and significant correlation was observed with the Rain variable (0.28).

For DMC, there were no significant correlations between climatic variables and environments grouped in the first and second analytic factors. However, a positive correlation was identified between the third factor and Tmax (maximum temperature) with a correlation coefficient of 0.26, while a negative correlation was found between the fourth factor and the same variable (-0.32). Additionally, Tmin (minimum temperature) showed a positive correlation with environments in the fourth analytic factor (0.30). Overall, temperature and precipitation variables had the most significant effect on the G×E in the first and second factor environments, while temperature alone contributed to explaining performance differences of clones in the third and fourth factor environments for the four analyzed productive traits.

### 3.10. Comparative analysis of cassava stability for dry root yield: FA vs. AMMI vs. GGE Models

To compare the stability and adaptability of cassava genotypes, we focused exclusively on the most important agronomic trait in selection, namely dry root yield (DRY). The *FA*_4_ model explained ~87% of the variance, while the application of the AMMI model for dissecting G×E interactions (S5 Table) revealed that the first four terms of AMMI were significant, elucidating 49.3% of the G×E. Additionally, GGE biplot analysis of cassava genotypes unveiled that the first two principal components explained 69.06% of the total G×E variance (S7 Fig) for this same trait.

In general, the AMMI model with the first and second multiplicative terms aptly cross-validated yield variation explained by G×E (S6 Fig). Genotypes with larger IPCA1 scores, regardless of positive or negative signs, exhibited higher interactions (unstable), while those with IPCA1 scores closer to zero were considered stable. Thus, genotypes BR12-107-002, BR11-24-156, Vassoura Preta, and BRS Verdinha displayed relatively smaller IPCA1 scores for DRY, indicative of stability and broader adaptation, whereas BRS Novo Horizonte, Corrente, Eucalipto, and BRS Gema de Ovo demonstrated higher IPCA1 scores (S6 Fig). According to AMMI’s top three selections, genotypes BR11-24-156, BRS Caipira and Vassoura Preta proved desirable for both favorable and unfavorable environments, as they include genotypes that maintain yield stability even with environmental improvements. Conversely, the new genotypes BR11-34-41, BR11-34-45, BR11-34-64, and BR11-34-69, which have intermediate IPCA1 values, were better suited to favorable conditions. In contrast, BRS Poti Branca, BRS Dourada, BRS Gema de Ovo, Correntão, and IAC-90 performed well in unfavorable environments. The use of the AMMI model to select these genotypes in their respective environments underscores their optimal adaptation.

In the GGE biplot analysis, genotypes BRS Novo Horizonte, BR11-34-41, BR11-34-69, BR11-34-45, and BR11-34-64 emerged as the highest yielding, while Eucalipto, IAC-90, BRS Gema de ovo, Cigana Preta, and Correntão were considered the least productive among vertex genotypes (S7 Fig). The ideal genotype, exhibiting the highest PC1 score (mean performance) and low G×E, was BRS Tapioqueira, BR11-34-69 and BR11-24-156, making it the most stable across diverse environments. Furthermore, BRS Caipira, BR11-34-45 and BR11-34-64 genotypes were closer to the ideal genotype and deemed desirable.

Regarding the FA model, genotypes BR11-34-41 (9.36 t ha^-1^), BR11-34-69 (9.32 t ha^-1^), BRS Novo Horizonte (9.23 t ha^-1^), and BR11-34-45 (9.17 t ha^-1^) exhibited the highest overall performance for DRY. In contrast, genotypes Corrente and BRS Poti Branca showed the lowest average performance for this trait, specifically 7.14 t ha^-1^ and 7.52 t ha^-1^, respectively.

Line regression slopes close to zero are typical of genotypes with stable yields or minimal variation across environments, indicating low G×E interaction. The FA model effectively identified groups of genotypes with these characteristics, as well as those demonstrating greater adaptability to specific environments based on their responses to changes in factor loadings. For example, genotype BR11-34-41 consistently showed a positive β₁ < 1 across the second to fourth factors, signifying significant stability in at least 37.7% of environments categorized within these factors (with loadings > 1.5) (Figs 6 and S4). Additionally, genotypes BRS Poti Branca, BRS Novo Horizonte, BR11-34-69, and BR11-34-64 (0.08 < β₁ < 0.68) exhibited high yield stability across a wide range of both favorable and unfavorable environments. Conversely, a positive β₁ > 1 suggests genotypes that are more responsive to environmental changes. Genotypes BR11-34-45 and Corrente demonstrated greater adaptation to environments with positive loadings for FA2 and FA4, respectively. However, these genotypes showed less responsiveness to environmental improvements in environments with negative loadings for these factors.

One advantage of the FA model over GGEbiplot and AMMI is its ability to capture a greater variance in G×E interaction, allowing for the classification and selection of superior genotypes based on regression line behavior. For instance, genotypes BR11-34-41 and BR11-34-69 displayed an ascending trend across environments grouped by *FA*_1_, while genotypes Cigana Preta and Corrente exhibited a declining trend, indicating unfavorable responses to these environments. Similar patterns were observed for other traits (FRY, ShY, and DMC), as illustrated in S6 and S7 Figs.

## 4. Discussion

### 4.1. Parameters and genetic gain in the selection of the best cassava genotypes

Cassava breeding programs conduct numerous METs annually to capture the diversity of the target population of environments (TPEs) across various production regions. The primary objective is to evaluate the performance of cultivars in these environments to meet the diverse needs of end-users. These needs include cultivars that are more productive, resistant to pests and diseases, have high nutritional quality, are well-adapted to the target regions, and exhibit high yield stability. The process of developing new cassava cultivars is lengthy, taking approximately 10 years, and is significantly influenced by G×E interactions. These interactions are complicated by increasingly unpredictable soil and climatic conditions, which interact with the genotype throughout the selection process. Understanding and exploring the nature of G×E interactions in cassava has been a challenging focus of many studies. In this context, our study aimed to explore analyses that can enhance the understanding of G×E interactions, with the goal of optimizing the selection process while considering the practical realities of cassava breeding programs.

Deciphering the complexities of G×E interactions in cassava breeding has long posed a significant challenge for researchers. This challenge is intricately linked to the diverse range of Testing and Evaluation Platforms (TPEs) [37]. Nevertheless, studies have shed light on the pivotal role played by the heterogeneity of genetic variance and covariance in the selection process by explaining the genetic correlations between trials. This helps capture environment-specific genetic effects and aids in studying cultivar adaptation and understanding the nature of G×E interactions [16,28]. Additionally, it is essential to explore the genetic correlations within METs trials to observe the diversity and connectivity between trials and mega-environments. These insights are instrumental in refining selection strategies, optimizing experimental designs, and tailoring cultivar recommendations to specific environments and needs.

The traits FRY, ShY, DRY, and DMC are considered crucial in the development of new cassava cultivars for industrial purposes, but their genetic estimates can be strongly influenced by the environment, reducing reliability [38]. Our results demonstrate that, in general, the genetic parameters differed significantly across the individual trials for all traits. For example, DMC exhibited the greatest amplitude of $\hat{H}²$, varying from 0.01 in the 2020.EP.GS.RA1 environment to 0.96 in the 2019.ERU.M.UFRB environment. However, in over 70% of the evaluated trials, notably high heritabilities (with an average of 0.66) were observed for all traits. According to [39], understanding heritability estimates can assist breeders in defining selection strategies, ensuring that genetic information from selected parents is passed on to their progenies through hybridization, thus allowing for greater genetic gain per selection cycle. On the other hand, unexplained variance due to G×E reduces heritability and expected genetic gains [40].

The joint analysis of various environments unveiled that $\hat{H}²$ indicates that the majority of genetic variability is not linked to the primary genetic effects, considering low values of $\hat{H}²$, ranging from 0.15 for ShY to 0.31 for DMC. This suggests that factors beyond the main genetic effects significantly contribute to the observed genetic variability across different environments. This variation may be attributed to the analysis of genotypes at different stages of the breeding program, as well as the use of data from experiments with varying plot sizes, number of repetitions, and different evaluation sites (S3 Table).

The $\hat{H}²$ estimates for yield in the advanced trials (EA) and uniform trials (ERU) were generally higher compared to the early-stage trials, such as the clonal trial (EC) and preliminary trial (EP) (S1 Fig). For instance, the average $\hat{H}²$ for the advanced trials was 0.70, while it was 0.55 for the preliminary production trials in terms of FRY. Similar trends were observed for other traits analyzed in individual trials. These results can be explained by the high heterogeneity of the initial trials in the cassava breeding program, which typically involve a smaller number of plants [41]. Thus, our findings suggest that advancing clones to trials with a larger number of plants in experimental plots and a greater number of environments becomes necessary to minimize the effects of G×E.

Similar findings were reported by [42] when comparing the genetic parameters obtained from clonal evaluation trials (CET) and preliminary trials (PYT) for yield in cassava, considering 23 full-sibling families (F_1_) and six self-pollinated families (S_1_). According to the authors, greater genetic gains were observed in the PYT trials compared to the CET trials. For example, the heritability increased from 0.30 to 0.88 for FRY and from 0.23 to 0.88 for DRY in both F_1_ and S_1_ families. Additionally, the coefficient of residual variation (CVe) was higher in the CET trials compared to the PYT trials for both FRY and DRY in F_1_ and S_1_ families. These results demonstrate the significant environmental influence in the initial trials.

For quantitative traits, heritability values above 0.40 are considered of medium to high magnitude, indicating easier transfer of quantitative inheritance traits to progenies. In the case of cassava, previous reports in the literature have shown heritability values ranging from 0.45 to 0.56 for DMC in multi-environment trials [35,38]. For FRY and DRY, heritability values ranging from 0.21 to 0.15 have been reported [43], which is similar to the heritability values obtained in the present study (0.20 and 0.18 for FRY and DRY, respectively).

Regarding the coefficient of genetic variation (*CVg*), it was consistently lower than the coefficient of residual variation (*CVr* <1.0) across the traits evaluated, ranging from 0.76 for ShY to 0.90 for FRY. This suggests that the variation attributed to genetic factors is lower than the variation due to environmental factors. Such a scenario indicates that relying solely on the selection of clones based on a limited number of environments may not be reliable, as the observed phenotypic variation is predominantly driven by environmental factors rather than genetic factors. Only DMC exhibited a *CVg*/CVr ratio greater than 1.0, indicating that the genetic variation exceeds the residual variation for this trait. This finding suggests that selection based on DMC could lead to more reliable genetic gains compared to other traits evaluated in the study.

Several studies have demonstrated the predominance of additive genetic effects in the expression of DMC in cassava [44–46]. Other studies have reported similar $\sigma_{g}^{2}$ values of 5.26 and below for joint trials in cassava, with average yields ranging from 34.22% to 24.12%, which is lower than the average yield in the present study (35.63%) [35,47]. These results suggest that the lower G×E for DMC allows, for maximizing the probability of selecting of more stable clones for this trait across different years and growing locations.

### 4.2. Using the FA model to explore G×E interaction in cassava

The analytic factor structure (*FA*_*k*_) is a valuable tool for dealing with unbalanced data and low connectivity between trials [16]. In the present study, despite genotypes being evaluated in at least 50% of the environments, the *FA*_4_ model explained more than 85% of the genetic variance for all four traits, indicating that the structure of the G×E interaction was well captured by the *FA* model.

METs used to assess G×E are costly because they require conducting trials in different locations/regions to observe the effects of soil and climate conditions on phenotypic expression. By exploring genetic correlations between pairs of environments, the FA model helps to minimize the effects resulting from G×E interaction and identify similar and contrasting environments. As can be seen, there is a high correlation between observed and predicted means in untested environments based on the FA model, as indicated by low scatter in the biplot. [48] reported 85% of genetic variance explained by *FA*_2_ when evaluating different levels of unbalance (10%, 30%, and 50%) in maize breeding trials.

These results can be attributed to the predictive ability of the *FA* model in modeling the variance matrix and genetic covariance pairs (VCOV) of genetic and residual effects across environments in METs trials. By considering the information from correlated environments and the effects of genotypes across trials, the *FA* model integrates and simplifies the estimation of environment-specific genetic variance and pairwise covariances. This approach approximates unstructured VCOV models and reduces the number of estimated parameters. The *FA* model also provides estimates of predicted genetic values for missing environments, genotypic scores, and environmental loadings. It maximizes the common variance among correlated factors by reducing the variables into a few latent factors that are related to the G×E, even in the presence of low connectivity between trials [9,26].

The use of the *FA* model in exploring G×E with historical cassava data is groundbreaking and opens up new possibilities for analysis, aiding breeders in decision-making regarding the recommendation of new genotypes and experimental designs. A similar study conducted by the International Institute of Tropical Agriculture (IITA) in Africa used the *FA* model to assess the stability and performance of 96 cassava varieties for fresh root yield across 48 UYT. This study found that 79.0% of the total genetic variation was captured by the FA3 model [49]. *FA* models have been employed by other researchers in clonal propagation species such as eucalyptus and some forest species to investigate adaptability and yield stability. All of these studies demonstrated that models capable of accommodating heterogeneous variances and covariances outperform traditional models, leading to greater genetic gains. By capturing the complexity of G×E interactions, FA models enable more accurate predictions of genotype performance, especially in environments with diverse conditions, and provide breeders with more reliable tools for selecting superior genotypes.

Due to the complexity of METs trials, it is crucial to use robust analysis methods that can handle heterogeneous variances and covariances between environments. Less robust methods may lead to biased genetic parameter estimates, unstable model fits, and failure to converge. In the present study, the *FA*_4_ model, with an acceptable percentage of variance explained, proved to be the most parsimonious for all four traits. The model selection criteria, such as AIC, and the number of estimated parameters (<253.0) were considered, ensuring reliable estimation of variance parameters. The accuracy of the model (*rFA*_4*M*_) was high (>90%), indicating a good fit. Similar model selection values favoring the *FA*_4_ model were reported in cotton [20].

The loadings of an environmental factor represent the proportion of genetic covariance between environments explained by that specific factor. This information allows for optimizing the number of environments in METs trials by selecting environments with high representativeness and identifying mega-environments based on environmental loadings. For example, in the case of DMC, the highest loads ranged from 1.03 (2021.EP.WX.RA1) to 2.81 (2021.EA.GS.AL), with 29 environments exhibiting genetic variance above 64% (*FA*_1_].

A similar pattern was observed for the other traits, although with variance values ≤50% (*FA*_1_). The first factor explained a substantial proportion of the genetic variation in G×E, indicating that the latent regression in the first factor had the greatest impact on the predicted genetic values of the genotypes. For instance, in the case of FRY, considering the average environmental loading [2.97], 40% of the environments exhibited positive loadings above the average, suggesting the presence of a mega-environment. Clustering these environments is recommended because there is less G×E, leading to FRY values exceeding 24 t ha^-1^ in this factor. Conversely, opposite directions of environmental loadings and genotypic scores demonstrate the influence of G×E on genotype performance and the clustering of trials based on environmental contrasts. High and positive loadings indicate environments with high discriminatory power and the ability to explain most of the observed genetic variation, as exemplified by 2019.EC.GS.UFRB environment (loadings of 7.48) for ShY. Low loadings, on the other hand, suggest environments that contribute little to the genetic variance across environments, as exemplified by 2018.ERU.UFRB environment (loading of 0.35) for the trait DRY.

In cases of positive correlations, the regression line between two hypothetical genotypes does not cross, and therefore the ranking of genotypes does not change between environments on that factor, representing an uncrossed G×E. This means that the response of genotypes to the trait does not vary across environments that are positively correlated with the factor. For example, genotype BR11-34-69 (-2.84) will exhibit a higher shoot yield response in the environment 2021.EP.WX.RA1 (-1.58) with a high loading and the same direction, indicating a positive correlation. On the other hand, negative loadings suggest heterogeneity of correlations across environments, indicating a tendency for a crossed G×E. This means that environmental responses are not equal across environments, resulting in rank shifting and specific adaptations that can be exploited. For example, genotype BR11-34-41 with *β*_1_>1 can be recommended for approximately 20 environments in the set of *FA*_1_ and *FA*_2_ (negatively charged) environments for FRY.

For environments with positive loadings, genotypes with negative scores tend to exhibit a reduction in the productive performance. Conversely, genotypes with positive scores, in the same direction as the environment, will have a favorable performance. For example, in the case of DRY, genotype BR11-34-41 with *β*_1_= 1.30 and the highest environmental load for the first factor from the environment 2020.ERU.UFV (16.53) both have positive values. This indicates a high agronomic performance of this genotype in that specific environment. However, for Corrente (*β*_1_ = -1.92), there will be a decrease in DRY, given the negative score for this environment in that specific factor. Conversely, for environment 2021.EC.GS.UFRB (*β*_1_ = -1.74), there will be a higher DRY. A similar pattern can be observed for the other traits.

In the study conducted by [17] on five commercial wheat varieties, the use of latent regression plots with the *FA*_5_ model explained 82% of the genetic variance. The *FA*_1_ model exhibited only positive loadings, suggesting that genotypes with positive slopes (*β*_1_) for this factor are desirable, and the predicted genetic values tend to increase for the set of environments with high estimated loadings.

### 4.3. Stability analysis of high-yielding genotypes in multi-environments by FA, AMMI e GGE

The use of the *FA*_4_ model in the cassava breeding program has proven to be highly effective in capturing a significant portion of the genetic variation for key traits such as FRY, ShY, DRY, and DMC. With percentages ranging from 87.31% to 95.52%, the model outperformed previous studies in *Pinus* species and sorghum, even when dealing with unbalanced data [12,16]. On the other hand, the AMMI and GGE methods captured a smaller proportion of the variance (~66.01% for four PCs), indicating that the use of models assuming homogeneity of variance in unbalanced data tends to penalize the main effects and capture more noise. This makes it more challenging to achieve greater model convergence and consequently reduces the precision in selection.

Overall, the criteria used for selecting stable and high-yielding genotypes were not consistent across the FA, AMMI, and GGE biplot models. However, the FA model showed superiority, particularly in capturing a larger proportion of the G×E interaction variance, identifying the degree of genetic connectivity among trials, exhibiting better correlations between trials, and providing more accurate estimates of genetic parameters such as heritability. These advantages enhance confidence in genotype selection across breeding cycles and in identifying new promising genotypes.

For example, the genotype BR11-34-69, with a *β*_1_ = 0.35, showed high FRY (33.65 t ha^-1^) in the *FA*_2_ environments, while the genotype BR11-34-45, with a *β*_1_ = 0.92, demonstrated stable performance for the trait ShY (25.80 t ha^-1^) in the same set of environments. However, these genotypes were identified as having specific adaptability to a different set of environments than those grouped by *FA*_2_ based on the AMMI and GGE methods. Conversely, a positive *β*_1_ > 1 suggests genotypes that are more responsive to environmental changes. For instance, BR11-34-41 and BRS Novo Horizonte displayed 9.36 t ha^-1^ of DRY and 38.04% of DMC, respectively, with *β*_1_ values of 1.30 and 1.18 for the set of environments in *FA*_1_. However, these genotypes were identified by the AMMI and GGE methods as having specific adaptability based on IPCA1.

These results suggest that the specific genetic variance for each environment and the different paired covariances between environments captured by the FA model allow for better estimation of genetic correlations between environments, leading to a more accurate grouping of correlated environments [11]. [49] reported that the FA model with the effect of G associated with G×E is more parsimonious compared to models that separate the genotype effect from G×E, such as the AMMI model. Additionally, [50] stated that the FA model can have a similar interpretation to SREG (sites regression-SREG, which is similar to GGE) if the genotype effect is not separated from the G×E interaction, or it can be similar to AMMI if the genotype effect is separated from the interaction (G + G×E). [51] observed some similarity between the AMMI2 and *FA*_2_ models. However, our results show that this similarity between models depends on various factors, including the magnitude of the G×E interaction. When comparing the predictive ability of the linear-bilinear model and mixed-effects models using the FA model, [49] found that the predictability of the model improved by up to 5–7% with FA for models with high complexity G×E and G + G×E effects, as is often the case in most MET trials in cassava. Thus, the main advantage of the FA model is its parsimony, its ability to accommodate heterogeneity of (co)variances, both residual and genotypic, and its capacity to handle incomplete and unbalanced data in the analyses. By considering genotypes as random effects, it also allows for the estimation of genetic values of the genotypes, which is essential for selection.

Thus, the latent regression plots provided valuable information. Genotypes suggested as stable reflected their response to the environmental covariate across all environments in the factor, including those that were not tested. These genotypes exhibited yield stability even with environmental improvements. Their stability is evident as they showed minimal response to changes in environmental loads, making them suitable for environments with low technological levels. On the other hand, the adapted genotypes are recommended for environments with high technological levels as they responded to increased environmental loads. Overall, the *FA* model facilitated a better understanding of the G×E by considering both the performance and stability of the evaluated genotypes. On the other hand, the GGE biplot model is particularly effective for visualizing and identifying mega-environments, as well as for selecting representative and discriminative environments [34,52]. In contrast, AMMI analysis enhances breeders’ ability to identify superior environmental conditions for exploiting specific adaptability, as well as for selecting and recommending optimal cultivation sites [53]. Depending on the specific needs and objectives, these methods can be used complementarily, thereby expanding breeding strategies and enhancing the understanding of the underlying parameters of the G×E interaction. This complementary use provides a more robust approach to addressing the complexities of genotype-environment interactions and improving crop performance across diverse conditions.

### 4.4. Correlation and identification of environments groups for regionalized recommendations

The G×E plays a significant role in genotype performance, particularly in the presence of challenging edaphoclimatic conditions that affect experimental trials. The *FA* model offers a valuable framework to explore the genetic correlations between environments and incorporate this information into the analysis. By doing so, it becomes possible to assess the effect and contribution of each environment on genotype performance and G×E through covariance, leading to the identification of specific groups of environments with high connectedness.

Environments that exhibit high environmental loading within each factor generally display strong correlations with one another, indicating minimal G×E. This demonstrates the effectiveness of factor rotation in grouping environments based on their similarities. Environments that are close to each other in a biplot tend to elicit similar biological responses among genotypes in that particular sector [54]. The *FA* model has been successfully used in maize to identify subgroups of genotypes/environments without G×E, allowing for the modeling of groups with consistent associations between environments [55].

High and positive correlations are advantageous in selecting new varieties as they minimize G×E and indicate the adaptability of genotypes in specific groups of environments, known as mega-environments. Mega-environments are characterized by homogeneous environmental conditions and similar performance of genotypes over time [56]. Consequently, environments with high and significant correlations can be grouped together or even discarded, while new environments can be included to identify environmental patterns and maximize genetic gains.

The heatmap generated by the *FA* model represents the pairwise matrix of genetic correlations between environments, revealing the magnitudes and directions of these correlations. In the present study, the correlations for the four traits ranged from medium to high magnitude. This indicates that more than 75% of the correlations showed similar performance among genotypes, suggesting the existence of clones that are adapted to regions with distinct climate and soil conditions. For example, FRY exhibited consistent genetic responses across most environments, except for 2020.ERU.NH2B, 2020.ERU.RA1, and 2021.ERU.NH3, which displayed high negative and significant correlations with the majority of evaluated environments. This suggests potential differences in genotype performance based on climatic conditions from one year to the next, likely influenced by the low and negative loadings (<-5.73) for *FA*_2_ and *FA*_3_, indicating the impact of the environmental covariate [Rain] on genotype performance in these environments.

The observed correlations imply that the influence of crop years (which reflects climatic variables) on the traits DRY and DMC was less pronounced compared to the effect of locations (which represents edaphic conditions). This finding aligns with previous research by [17], indicating that most G×E occurs across different years. However, for FRY and ShY, both year and site effects were significant, highlighting the complexity of selecting genotypes for these traits. It is noteworthy that the inclusion of a large number of years (>5 years) in this study, representing a random sample of regional weather stations, enhances the reliability of predicting environmental effects and facilitates the recommendation of genotypes.

The heterogeneity of variance and the proportion of additive variance strongly depend on the specific trait and the correlations between environments. In this study, the high proportion of positive correlations between pairs of environments, combined with the heritability of the traits, enhances the effectiveness of the *FA* model in explaining the substantial genetic variance and identifying superior genotypes and environments with low G×E [57].

### 4.5. Environmental variables and their relationship with the G×E interaction

To enhance the understanding of the G×E and provide more consistent and biologically meaningful results, we employed a comprehensive approach. First, we obtained environmental loadings (obtained by the analytic factor structure, *FA*_4_) which, on their own, can be challenging to interpret. To overcome this limitation, we correlated these loadings with potentially informative environmental covariates. By incorporating edafoclimatic variables, we aimed to discern the temporal influence of the environment on genotype performance. This allowed us to differentiate between the effects of climate, soil, and their interactions, thereby characterizing the environment by correlating it with environmental loadings [17,30,58]. Indeed, this aspect is fundamental, given that the influence of climatic seasonality, coupled with the genetic constitution of genotypes across different trials and years, poses challenges in predicting G×E interactions. Therefore, supplementary information about the environments becomes important [37]. This highlights the necessity of incorporating comprehensive environmental data and genetic information to enhance the accuracy of genotype-environment interaction assessments, thereby facilitating more informed breeding decisions.

Overall, we found significant correlations, ranging from 0.26 to 0.58, between climate variables and environmental loads. However, none of the climate variables could account for more than 58% of the variance in the G×E. These findings suggest, for example, that the *FA*_1_ loadings capture a significant portion of its variability, hinting at a potential sensitivity to variations in those factors. In other words, precipitation, solar radiation, and altitude were the environmental covariates that most explained the genetic variability of genotype BR11-34-69 for FRY, impacting adaptability and stability across the grouped environments related to this factor.

Notably, maximum daily temperature consistently demonstrated a strong association across all four analytic factors (0.17 to 0.32) for the DMC trait. For instance, the BRS Novo Horizonte variety exemplifies this relationship. This genotype exhibited higher DMC (38.04%) and *β*_1_ values of 1.18 and 1.30 for *FA*_1_ and *FA*_3_, respectively, indicating a greater sensitivity to high temperatures. However, the significance of the correlation *FA* × environmental covariable, was observed primarily for the environments allocated in *FA*_3_ and *FA*_4_ (~25 environments). On the other hand, rainfall was the most influential covariate for three analytic factors (0.15 to 0.58), specifically significant for *FA*_1_ and *FA*_2_ of the FRY trait. In their analysis, [59] reported correlations between mean temperature and *FA*_3_ loads spanning from -0.28 to 0.65. This correlation contributed substantially, accounting for 27.8% of the G×E in *Picea abies* progenies’ performance. This underscores the significance of environmental covariates in shaping both the level and pattern of G×E, offering the potential to forecast genotype performance by correlating historical climate data with *FA*.

These findings corroborate the influence of edaphoclimatic conditions on the stratification of G×E in cassava, making it more predictable. For example, the transition from the dry period to the onset of rainfall triggers a process of starch reallocation from the roots to support the growth of the above-ground parts [60]. Conversely, low temperatures and insufficient solar radiation can hinder vegetative growth, thereby affecting the production of above-ground biomass and consequently starch production [61]. Environments that do not meet these minimum conditions tend to penalize genotypes in terms of productivity and further influence the G×E. These results underscore the significant impact of reducing essential productive and quality attributes of cassava roots, leading to a subsequent decline in starch yield.

The identification of climate variables associated with environmental loads in the *FA* model provides valuable insights into the factors that influence the classification of genotypes regarding the G×E in cassava. As highlighted by [49], the *FA* model’s ability to utilize genetic correlations between environments enhances predictability by up to 6% compared to models assuming homogeneity of variance between environments. Predicting the various factors that impact root yield is essential, as climatic factors strongly influence the goals and outcomes of breeding programs [60].

Lastly, we emphasize that the utilization of the METs dataset from the cassava breeding program, coupled with the analysis of climate variables within the *FA* model framework, can greatly assist in identifying genotypes with stability and high performance across diverse environments. It can also expand the scope of predicting transient components that influence the G×E beyond the tested environments. Furthermore, this approach allows for capturing the repeatability and trends in performance and stability over the years, provided that comprehensive sets of information on environmental covariates from different years and locations are grouped together to enhance the reliability of studying the G×E for recommending new genotypes.

### 4.6. Future prospects

In the context of global warming and climate change, cassava’s role in ensuring food security becomes increasingly important. Consequently, the prospects for genetic improvement of cassava, particularly through the analysis of adaptability and stability using the multiplicative mixed model of the analytic factor, are crucial in developing high-performing genotypes for both tested and untested environments. The METs trials, being highly unbalanced due to the introduction of new genotypes (uniform and advanced trials) and the removal of low-performing genotypes, are complex management throughout Brazil.

There are several statistical approaches to explore the GxE interaction. However, models that use the *s*×*s* matrix structure of genetic variance and covariance components among the *s* evaluated environments provide a better understanding of the G×E interaction and the genetic architecture of breeding traits, along with estimates of all genetic environment-environment correlations. Currently, the most parsimonious approach to modeling the genetic variance-covariance matrix is based on the FA structure [14,51], which also allows for its extension to estimate additive and non-additive effects simultaneously [62].

The flexibility in obtaining genetic parameters through the FA method offers various opportunities to enhance the understanding of the G×E interaction in cassava breeding programs. With the increasing practical use of genomic selection [20,63] and numerous genome-wide association studies [64,65], there are predictive advantages in these models when incorporating the G×E interaction for quantitative traits. Some examples of this are the studies by [49], which showed that FA models exhibited up to a 6% advantage in predictive accuracy compared to models that considered the same variance and correlation between environments; while [66] demonstrated that genomic selection models that take into account G×E had greater predictive capacity compared to models that ignore G×E. Therefore, it is intended to include information from FA models to update genomic prediction models and GWAS studies incorporating the G×E interaction.

Another avenue for enhancing the study of G×E interaction within cassava breeding programs is by employing *FA* models, which offer several potential improvements. One such enhancement is envirotyping, a method involving detailed environmental characterization to identify predominant environmental types or existing experimental networks. This enables the calculation of environmental variance-covariance, facilitating enviromic prediction to forecast breeding zones for current and future trials [37]. Recently, [67] introduced a novel predictive breeding approach called GIS-FA. This innovative method integrates geographic information system (GIS) techniques, *FA* models, partial least squares regression (PLS), and environmental data to predict phenotypic performance in untested environments. GIS-FA allows for the identification of new breeding scenarios in specific environmental groups, where genotypes demonstrate superior predicted performance, even in locations where they have not been tested. This approach offers significant advantages over traditional methods such as AMMI and GGE biplot in studying GxE interactions. Other opportunities for improving this study include incorporating the pedigree matrix into the *FA* model, which provides a broader understanding of genetic effects on agronomic performance. This allows for the identification of the contributions of dominance and additive effects for target traits in cassava [12,26]. Additionally, to minimize potential selection bias and integrate knowledge from other fields, it is important to explore environmental covariates that may help predict the performance of cassava genotypes in untested environments within MET trials. The goal is for the genetic improvement program to become increasingly precise, leading to higher genetic gains and providing short- and medium-term potential. By integrating advanced predictive tools and more comprehensive data, cassava breeding can achieve greater efficiency and accuracy in selecting superior genotypes for diverse environments.

Furthermore, by obtaining historical climate data from different regions, it becomes possible to construct climate indices that aid in predicting the pattern of G×E and genetic values of untested clones. Our results suggest that incorporating environmental covariates into the FA model enhances its efficiency in understanding G×E interactions by capturing cross-environmental interactions and explaining a greater proportion of genetic variance. The FA model’s structure, as demonstrated in this study, emphasizes the importance of precipitation and temperature as key factors directly influencing the G×E. This integrated approach facilitates the development of effective strategies for cassava breeding programs, ensuring the selection of genotypes that exhibit enhanced adaptability and stability in varying climatic conditions. In this regard, the FA model’s structure proves invaluable in guiding cassava improvement programs, enabling the estimation of reliable and efficient genetic parameters. These innovative approaches warrant further exploration in future reviews, supported by new regional trial data.

## 5. Conclusion

This study reveals significant genetic variance in cassava, even in the analysis of a small number of genotypes at the final stage of selection. This indicates that productive data surpass those of local varieties, highlighting the potential for improving cassava yield through genetic selection. This was achieved by employing a robust environment-specific variance-covariance structure to ensure stable and convergent fits. To accomplish this, we utilized the analytic factor *FA*_4_, which exhibited the lowest AIC and explained over 87% of the total genetic variance. The genetic correlations, derived from the analytic factor structure (*FA*_4_), indicate distinct patterns of G×E interactions across four agronomic traits, which can guide more effective selection strategies for high-performing genotypes and the identification of stable, high-yielding mega-environments.

Additionally, the *FA* model captured more variance in G×E interactions compared to the GGE biplot and AMMI models, suggesting superior selection efficiency and genetic gains. The *FA* model also allowed us to predict genotype performance in environments where they were not tested, potentially reducing the costs of future trials. The genotypes BR11-34-69, BR11-34-45, and BR11-34-64 were identified as the most stable for three important traits, with potential for release as new cultivars. Another promising genotype, BR11-34-41, showed stable performance across multiple environments and demonstrated superior fresh root yield (FRY).

Furthermore, the relationship between FA₄ and climatic variables revealed that rainfall, altitude, and solar radiation were key factors influencing G×E interactions. Understanding these environmental covariates can help breeders optimize planting and harvesting schedules, based on the seasons with the greatest impact on cassava performance. This approach provides valuable insights into the complex G×E interactions in cassava, enabling breeders to select genotypes with better yield stability and adaptability to specific environmental conditions.

Future studies could incorporate the use of advanced envirotyping techniques and GIS-FA models to further predict genotype performance in untested environments. Additionally, incorporating pedigree data and exploring other environmental covariates may improve the precision of genotype predictions. Expanding these models to include long-term climatic data and exploring additional genetic interactions would refine selection strategies and increase the accuracy of breeding programs for cassava.

## Supporting information

S1 FigHeatmap plot of genetic parameters: Genetic ($\sigma_{g}^{2}$) and residual variance ($\sigma_{r}^{2}$), broad-sense heritability (*h*^2^) and coefficient of variation (CV%), for fresh root yield (FRY), shoot yield (ShY), dry root yield (DRY) and dry matter content (DMC), 22 cassava genotypes in 57, 56, 53 and 59 environments respectively environments.(DOCX)

S2 Fig Pearson’s correlation coefficients, the heritability (H^2^), experimental accuracy (Ac) and coefficient of variation (CV%) for fresh root yield (FRY-A), shoot yield (ShY-B), dry root yield (DRY-C) and dry matter content in roots (DMC-D), respectively. (DOCX)

S3 Fig Proportion of phenotypic variance for fresh root yield (FRY), shoot yield (ShY), dry root yield (DRY) and dry matter content (DMC) evaluated with 22 cassava genotypes in 57, 56, 53 and 59 environments, respectively. (DOCX)

S4 Fig Heatmap plot of environmental loadings after varimax rotation, for the four-factor analytical model (*FA*_4_) for several agronomic attributes in cassava, for fresh root yield (FRY), shoot yield (ShY), dry root yield (DRY) and dry matter content (DMC), 22 cassava genotypes in 57, 56, 53 and 59 environments respectively environments. (DOCX)

S5 Fig Boxplot of the phenotypic mean of the individual trials for the traits: A) fresh root yield (FRY), B) shoot yield (ShY), C) dry root yield (DRY) and D) dry matter content in roots (DMC) of cassava evaluated with 22 cassava genotypes in 57, 56, 53 and 59 environments respectively environments.
(DOCX)

S6 Fig Biplot of adaptability and stability values based of additive main effects and multiplicative interaction–AMMI for fresh root yield (A), shoot yield (B), dry root yield (C), and dry matter content in roots (D), evaluated in 22 cassava genotypes in multi-environment trials.
(DOCX)

S7 Fig Biplot of adaptability and stability values-based genotype main effects plus genotype × environment interaction effects–GGE for fresh root yield (A), shoot yield (B), dry root yield (C), and dry matter content in roots (D), evaluated in 22 cassava genotypes in multi-environment trials.
(DOCX)

S1 Table List of the cassava field trials evaluated from 2013 to 2021, for the four agronomic traits. (DOCX)

S2 Table Summary of the joint maximum likelihood ratio test analysis of 22 cassava genotypes evaluated in 57, 56, 53 and 59 environments for fresh root yield (FRY), shoot yield (ShY), dry root yield (DRY) and dry matter content in roots (DMC), respectively. (DOCX)

S3 Table Summary of genetic parameters of the joint analysis for fresh root yield of 22 cassava genotypes evaluated in 57, 56, 53 and 59 environments for fresh root yield (FRY), shoot yield (ShY), dry root yield (DRY) and dry matter content in roots (DMC), respectively. (DOCX)

S4 Table Selected genotypes based on predicted means (BLUP), and slope of the regression (β1) of the latent regression of the analytic model after varimax rotation. (DOCX)

S5 Table Analysis of variance by the additive main effects and multiplicative interaction (AMMI) for fresh root yield, shoot yield, dry root yield, and dry matter content in roots, evaluated in 22 cassava genotypes in multi-environment trials. (DOCX)
