# Supplementary material for: Genotype x environment interaction in cassava multi-environment trials via analytic factor
Source: PLoS One. 2024 Dec 9;19(12):e0315370. doi: 10.1371/journal.pone.0315370 (PMC11627386; doi:10.1371/journal.pone.0315370)
Supplement: S1 Fig — (DOCX) [file pone.0315370.s001.docx]

**
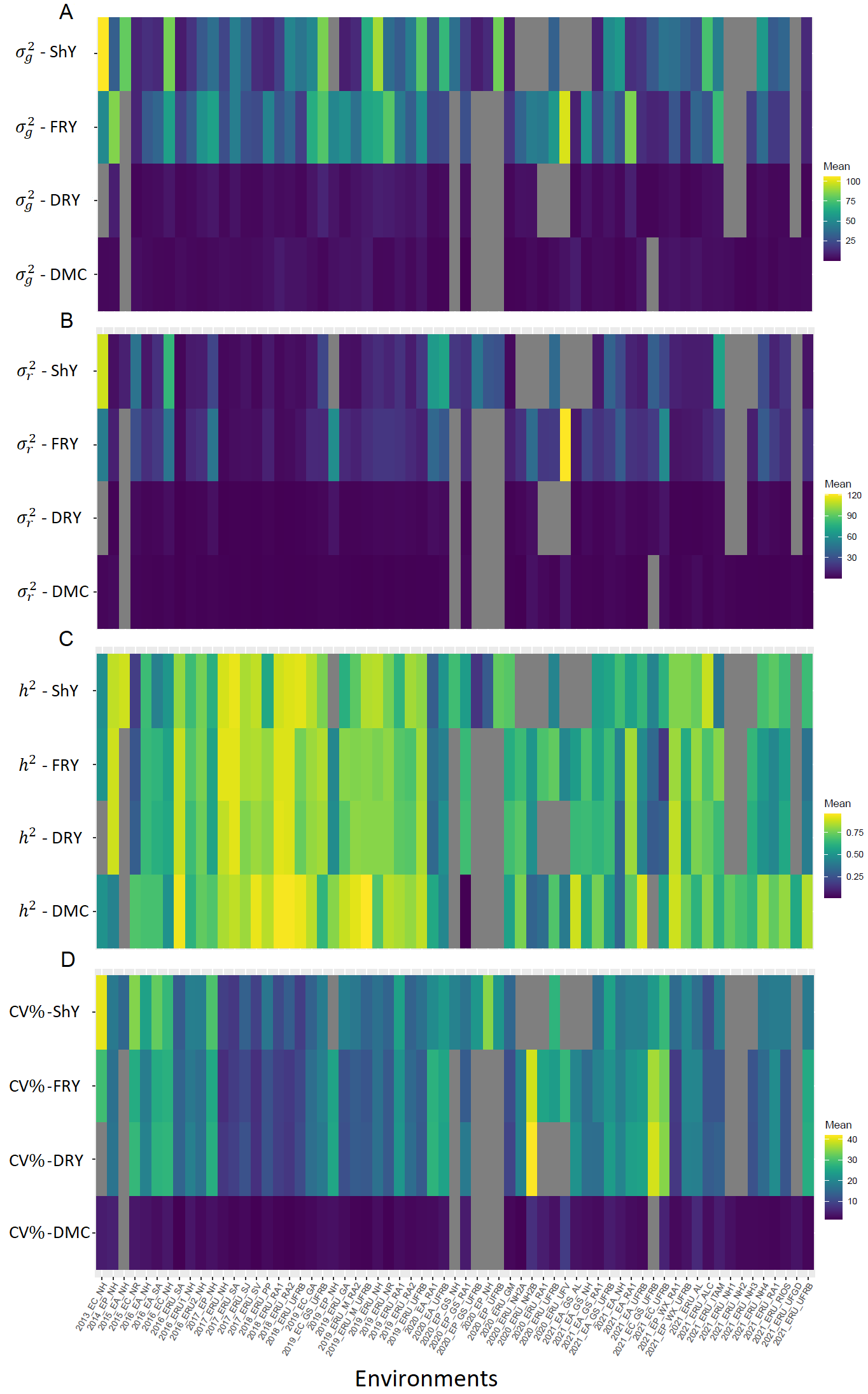
**

**Figure S1**. Heatmap plot of genetic parameters: genetic ($\sigma_{g}^{2}$) and residual variance ($\sigma_{r}^{2}$), broad-sense heritability ($h^{2}$) and coefficient of variation (CV$\%$), for fresh root yield (FRY), shoot yield (ShY), dry root yield (DRY) and dry matter content (DMC), 22 cassava genotypes in 57, 56, 53 and 59 environments respectively environments.
