# Supplementary material for: Genotype x environment interaction in cassava multi-environment trials via analytic factor
Source: PLoS One. 2024 Dec 9;19(12):e0315370. doi: 10.1371/journal.pone.0315370 (PMC11627386; doi:10.1371/journal.pone.0315370)
Supplement: S2 Fig — (DOCX) [file pone.0315370.s002.docx]

**
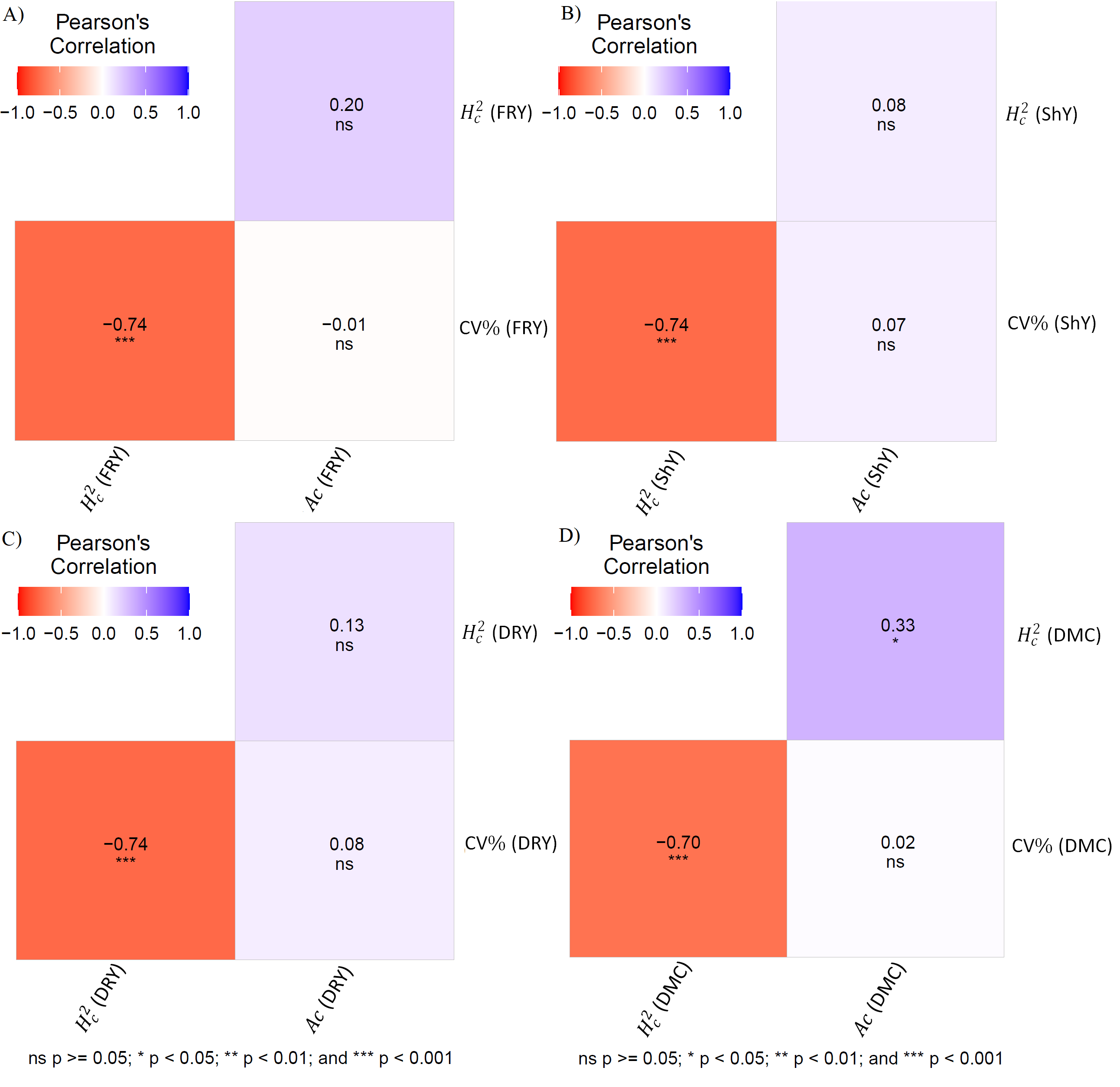
**

**Figure S2**. Pearson’s correlation coefficients, the heritability (H^2^), experimental accuracy (Ac) and coefficient of variation (CV%) for fresh root yield (FRY-A), shoot yield (ShY-B), dry root yield (DRY-C) and dry matter content in roots (DMC-D), respectively.
