# Supplementary material for: Genotype x environment interaction in cassava multi-environment trials via analytic factor
Source: PLoS One. 2024 Dec 9;19(12):e0315370. doi: 10.1371/journal.pone.0315370 (PMC11627386; doi:10.1371/journal.pone.0315370)
Supplement: S3 Fig — (DOCX) [file pone.0315370.s003.docx]

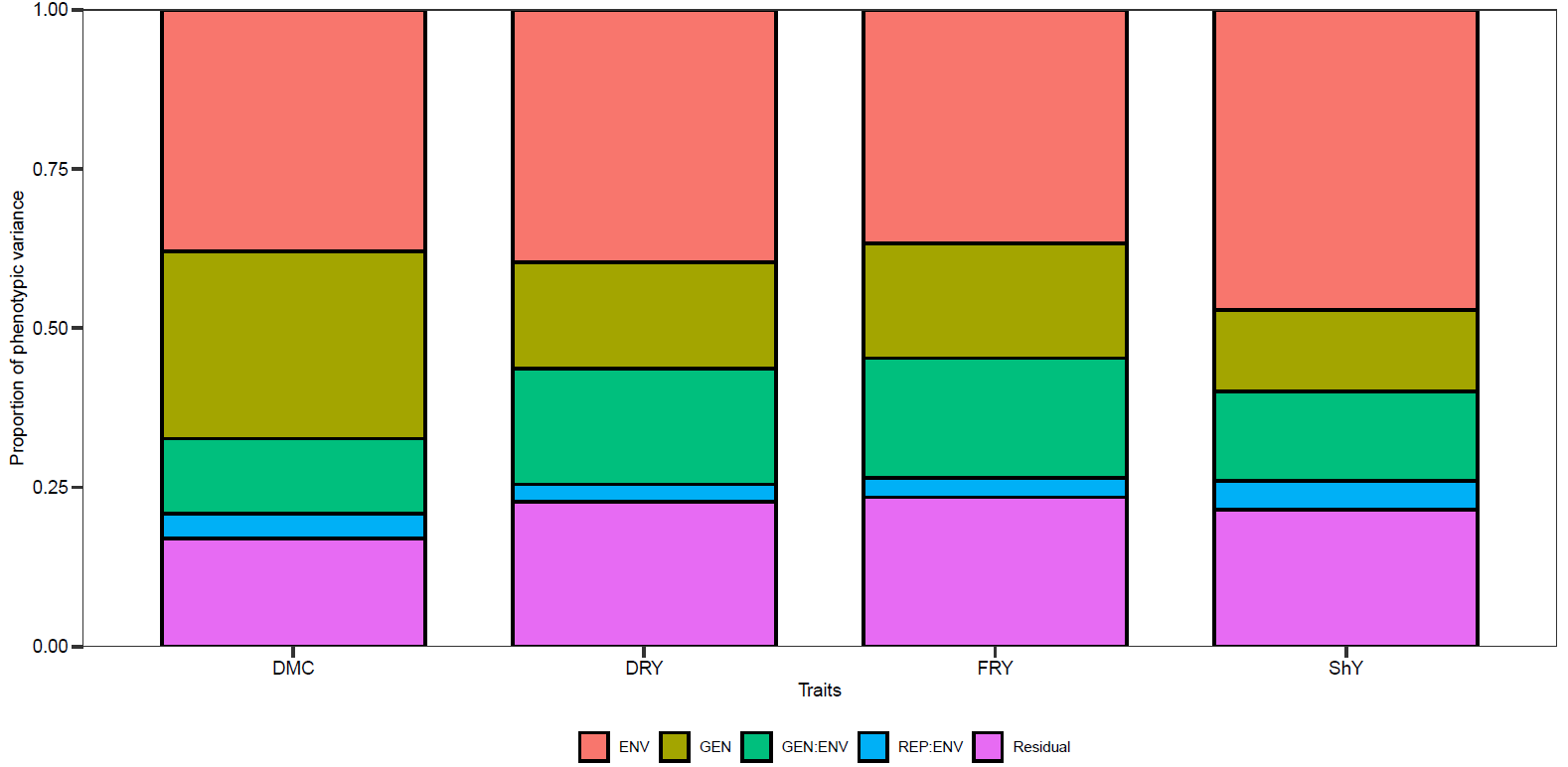


**Figure S3.** Proportion of phenotypic variance for fresh root yield (FRY), shoot yield (ShY), dry root yield (DRY) and dry matter content (DMC) evaluated with 22 cassava genotypes in 57, 56, 53 and 59 environments, respectively.
