# Supplementary material for: Genotype x environment interaction in cassava multi-environment trials via analytic factor
Source: PLoS One. 2024 Dec 9;19(12):e0315370. doi: 10.1371/journal.pone.0315370 (PMC11627386; doi:10.1371/journal.pone.0315370)
Supplement: S4 Fig — (DOCX) [file pone.0315370.s004.docx]

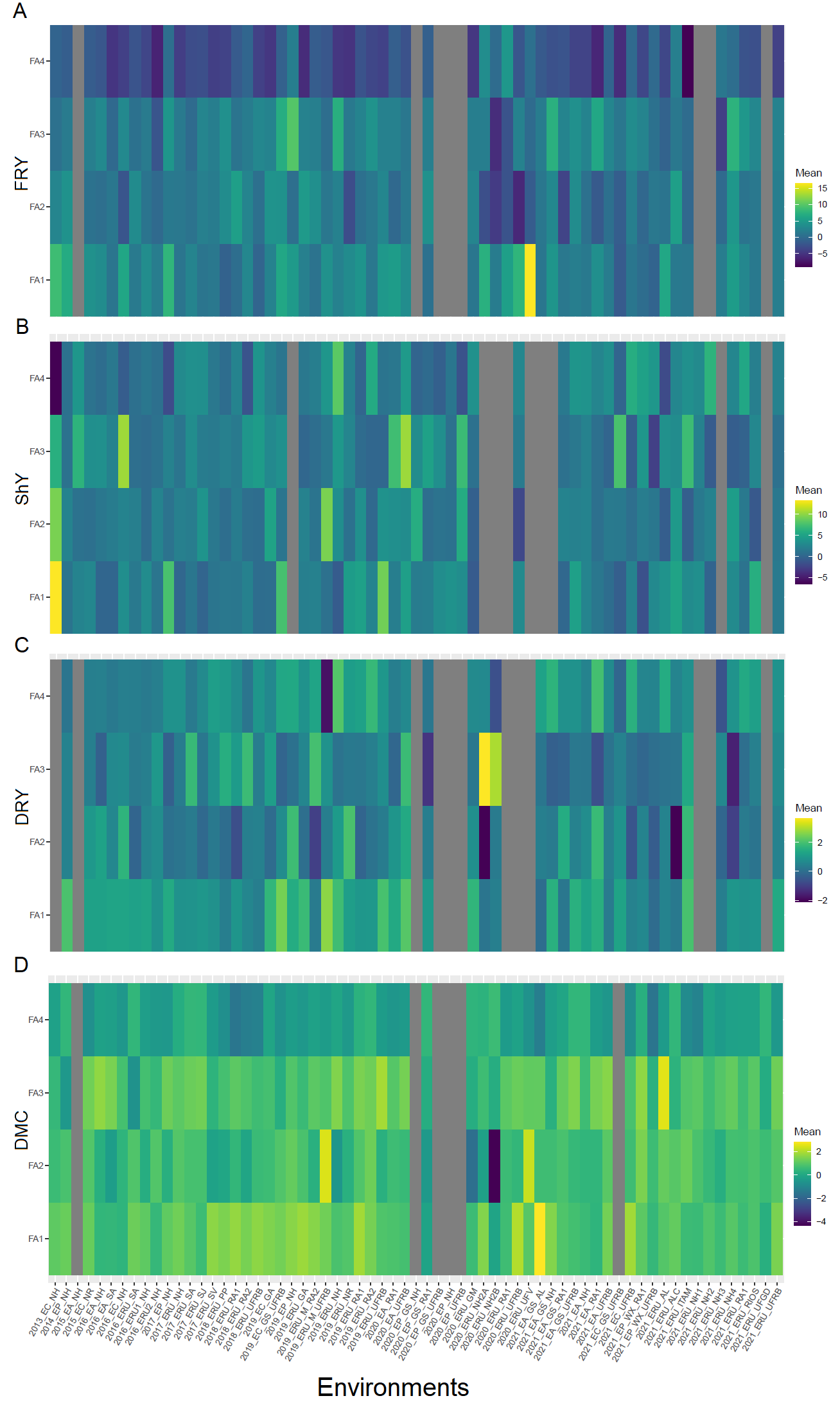


**Figure S4**. Heatmap plot of environmental loadings after varimax rotation, for the four-factor analytical model ${(FA}_{4})$ for several agronomic attributes in cassava, for fresh root yield (FRY), shoot yield (ShY), dry root yield (DRY) and dry matter content (DMC), 22 cassava genotypes in 57, 56, 53 and 59 environments respectively environments.
