# Supplementary material for: Genotype x environment interaction in cassava multi-environment trials via analytic factor
Source: PLoS One. 2024 Dec 9;19(12):e0315370. doi: 10.1371/journal.pone.0315370 (PMC11627386; doi:10.1371/journal.pone.0315370)
Supplement: S5 Fig — Boxplot of the phenotypic mean of the individual trials for the traits: A) fresh root yield (FRY), B) shoot yield (ShY), C) dry root yield (DRY) and D) dry matter content in roots (DMC) of cassava evaluated with 22 cassava genotypes in 57, 56, 53 and 59 environments respectively environments. (DOCX) [file pone.0315370.s005.docx]

**
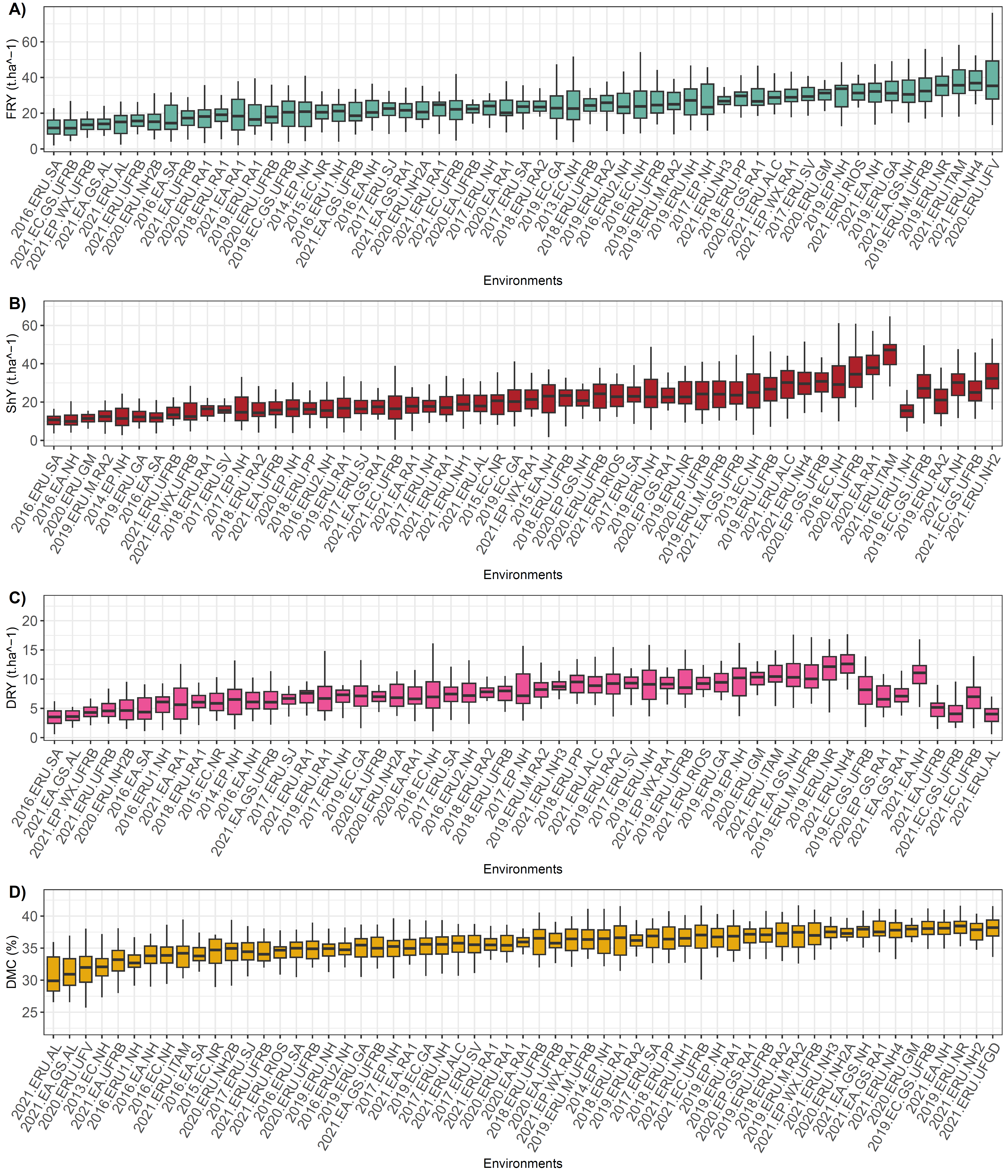
**

**Figure S5**. Boxplot of the phenotypic mean of the individual trials for the traits: A) fresh root yield (FRY), B) shoot yield (ShY), C) dry root yield (DRY) and D) dry matter content in roots (DMC) of cassava evaluated with 22 cassava genotypes in 57, 56, 53 and 59 environments respectively environments.
