# Supplementary material for: Genotype x environment interaction in cassava multi-environment trials via analytic factor
Source: PLoS One. 2024 Dec 9;19(12):e0315370. doi: 10.1371/journal.pone.0315370 (PMC11627386; doi:10.1371/journal.pone.0315370)
Supplement: S6 Fig — Biplot of adaptability and stability values based of additive main effects and multiplicative interaction–AMMI for fresh root yield (A), shoot yield (B), dry root yield (C), and dry matter content in roots (D), evaluated in 22 cassava genotypes in multi-environment trials. (DOCX) [file pone.0315370.s006.docx]

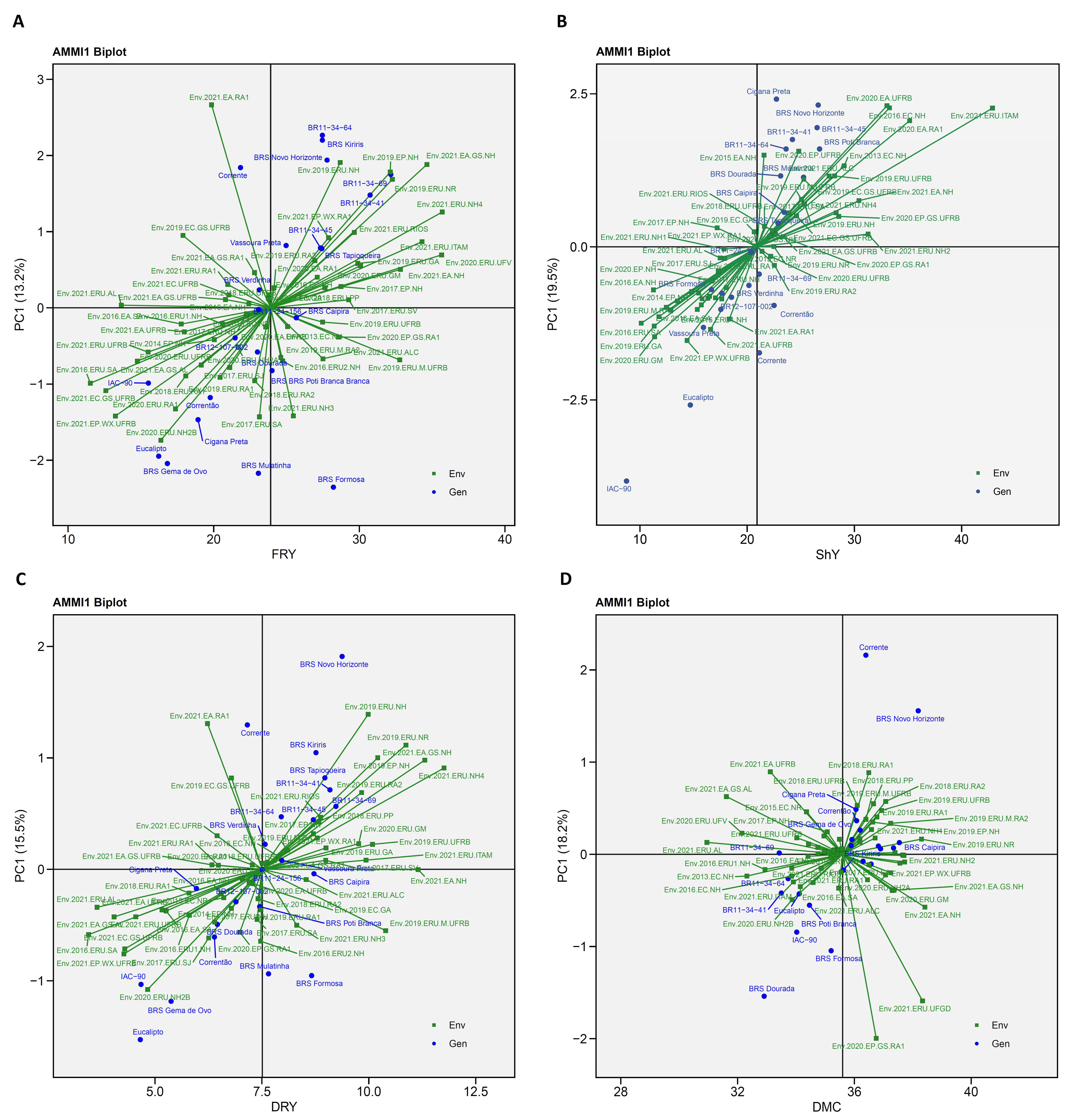


**Figure S6.** Biplot of adaptability and stability values based of additive main effects and multiplicative interaction – AMMI for fresh root yield (A), shoot yield (B), dry root yield (C), and dry matter content in roots (D), evaluated in 22 cassava genotypes in multi-environment trials.
