# Supplementary material for: Genotype x environment interaction in cassava multi-environment trials via analytic factor
Source: PLoS One. 2024 Dec 9;19(12):e0315370. doi: 10.1371/journal.pone.0315370 (PMC11627386; doi:10.1371/journal.pone.0315370)
Supplement: S7 Fig — Biplot of adaptability and stability values-based genotype main effects plus genotype × environment interaction effects–GGE for fresh root yield (A), shoot yield (B), dry root yield (C), and dry matter content in roots (D), evaluated in 22 cassava genotypes in multi-environment trials. (DOCX) [file pone.0315370.s007.docx]

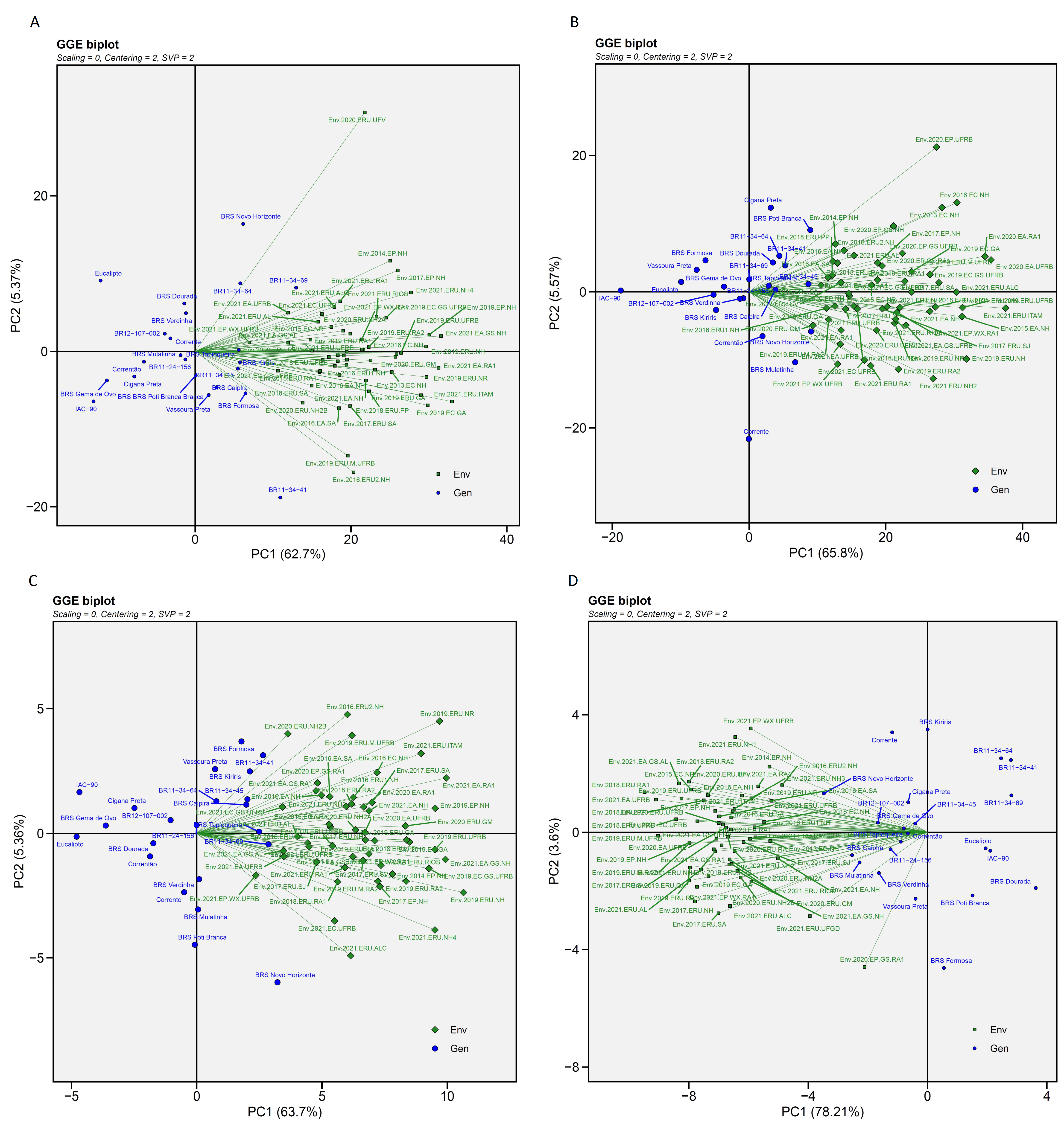


**Figure S7.** Biplot of adaptability and stability values-based genotype main effects plus genotype × environment interaction effects – GGE for fresh root yield (A), shoot yield (B), dry root yield (C), and dry matter content in roots (D), evaluated in 22 cassava genotypes in multi-environment trials.
