# Supplementary material for: Genotype x environment interaction in cassava multi-environment trials via analytic factor
Source: PLoS One. 2024 Dec 9;19(12):e0315370. doi: 10.1371/journal.pone.0315370 (PMC11627386; doi:10.1371/journal.pone.0315370)
Supplement: S1 Table — (DOCX) [file pone.0315370.s008.docx]

**Table S1.** List of the cassava field trials evaluated from 2013 to 2021, for the four agronomic traits.

| Trial | Year | Type of trial | Location | State |
| --- | --- | --- | --- | --- |
| 2013.EC.NH | 2013 | Clonal evaluation trial | Laje-Novo Horizonte | Bahia |
| 2014.EP.NH | 2014 | Preliminary yield trial | Laje-Novo Horizonte | Bahia |
| 2015.EA.NH | 2015 | Advanced yield trial | Laje-Novo Horizonte | Bahia |
| 2015.EC.NR | 2015 | Clonal evaluation trial | Valença | Bahia |
| 2016.EA.NH | 2016 | Advanced yield trial | Laje-Novo Horizonte | Bahia |
| 2016.EA.SA | 2016 | Advanced yield trial | Santo Amaro | Bahia |
| 2016.EC.NH | 2016 | Clonal evaluation trial | Laje-Novo Horizonte | Bahia |
| 2016.ERU1.NH | 2016 | Uniform yield trial | Laje-Novo Horizonte | Bahia |
| 2016.ERU2.NH | 2016 | Uniform yield trial | Laje-Novo Horizonte | Bahia |
| 2016.ERU.SA | 2016 | Uniform Yield Test | Santo Amaro | Bahia |
| 2017.EP.NH | 2017 | Preliminary yield trial | Laje-Novo Horizonte | Bahia |
| 2017.ERU.NH | 2017 | Uniform Yield Test | Laje-Novo Horizonte | Bahia |
| 2017.ERU.SA | 2017 | Uniform Yield Test | Santo Amaro | Bahia |
| 2017.ERU.SJ | 2017 | Uniform Yield Test | Laje São Jorge | Bahia |
| 2017.ERU.SV | 2017 | Uniform Yield Test | Laje Sombra Verde | Bahia |
| 2018.ERU.RA1 | 2018 | Uniform Yield Test | Laje Rio de areia 1 | Bahia |
| 2018.ERU.RA2 | 2018 | Uniform Yield Test | Laje Rio de areia 2 | Bahia |
| 2018.ERU.PP | 2018 | Uniform Yield Test | Laje Propriedade particular | Bahia |
| 2018.ERU.UFRB | 2018 | Uniform Yield Test | Cruz das Almas-UFRB | Bahia |
| 2019.EC.GA | 2019 | Clonal evaluation trial | Laje Gavião | Bahia |
| 2019.EC.GS.UFRB | 2019 | Clonal evaluation trial | Cruz das Almas-UFRB | Bahia |
| 2019.EP.NH | 2019 | Preliminary yield trial | Laje-Novo Horizonte | Bahia |
| 2019.ERU.GA | 2019 | Uniform Yield Test | Laje Gavião | Bahia |
| 2019.ERU.NH | 2019 | Uniform Yield Test | Laje-Novo Horizonte | Bahia |
| 2019.ERU.NR | 2019 | Uniform Yield Test | Valença | Bahia |
| 2019.ERU.RA1 | 2019 | Uniform Yield Test | Laje Rio de areia 1 | Bahia |
| 2019.ERU.RA2 | 2019 | Uniform Yield Test | Laje Rio de areia 2 | Bahia |
| 2019.ERU.M.RA2 | 2019 | Uniform Yield Test | Laje Rio de areia 2 | Bahia |
| 2019.ERU.UFRB | 2019 | Uniform Yield Test | Cruz das Almas-UFRB | Bahia |
| 2019.ERU.M.UFRB | 2019 | Uniform Yield Test | Cruz das Almas-UFRB | Bahia |
| 2020.EA.UFRB | 2020 | Advanced yield trial | Cruz das Almas-UFRB | Bahia |
| 2020.EA.RA1 | 2020 | Advanced yield trial | Laje Rio de areia 1 | Bahia |
| 2020.EP.GS.NH | 2020 | Preliminary yield trial | Laje-Novo Horizonte | Bahia |
| 2020.EP.NH | 2020 | Preliminary yield trial | Laje-Novo Horizonte | Bahia |
| 2020.EP.UFRB | 2020 | Preliminary yield trial | Cruz das Almas-UFRB | Bahia |
| 2020.EP.GS.RA1 | 2020 | Preliminary yield trial | Laje Rio de areia 1 | Bahia |
| 2020.EP.GS.UFRB | 2020 | Preliminary yield trial | Cruz das Almas-UFRB | Bahia |
| 2020.ERU.UFV | 2020 | Uniform Yield Test | Florestal-UFV | Minas Gerais |
| 2020.ERU.GM | 2020 | Uniform Yield Test | Governador Mangabeira | Bahia |
| 2020.ERU.NH2A | 2020 | Uniform Yield Test | Laje-Novo Horizonte-2A | Bahia |
| 2020.ERU.NH2B | 2020 | Uniform Yield Test | Laje-Novo Horizonte-2B | Bahia |
| 2020.ERU.UFRB | 2020 | Uniform Yield Test | Cruz das Almas-UFRB | Bahia |
| 2020.ERU.RA1 | 2020 | Uniform Yield Test | Laje Rio de areia 1 | Bahia |
| 2021.EC.UFRB | 2021 | Clonal evaluation trial | Cruz das Almas-UFRB | Bahia |
| 2021.EA.UFRB | 2021 | Advanced yield trial | Cruz das Almas-UFRB | Bahia |
| 2021.EA.NH | 2021 | Advanced yield trial | Laje-Novo Horizonte | Bahia |
| 2021.EA.RA1 | 2021 | Advanced yield trial | Laje Rio de areia 1 | Bahia |
| 2021.EA.GS.AL | 2021 | Advanced yield trial | Alagoinhas | Bahia |
| 2021.EA.GS.NH | 2021 | Advanced yield trial | Laje-Novo Horizonte | Bahia |
| 2021.EA.GS.UFRB | 2021 | Advanced yield trial | Cruz das Almas-UFRB | Bahia |
| 2021.EA.GS.RA1 | 2021 | Advanced yield trial | Laje Rio de areia 1 | Bahia |
| 2021.EP.WX.UFRB | 2021 | Preliminary yield trial | Cruz das Almas-UFRB | Bahia |
| 2021.EP.WX.RA1 | 2021 | Preliminary yield trial | Laje Rio de areia 1 | Bahia |
| 2021.ERU.UFRB | 2021 | Uniform Yield Test | Cruz das Almas-UFRB | Bahia |
| 2021.ERU.RA1 | 2021 | Uniform Yield Test | Laje Rio de areia 1 | Bahia |
| 2021.ERU.NH1 | 2021 | Uniform Yield Test | Laje-Novo Horizonte1 | Bahia |
| 2021.ERU.NH2 | 2021 | Uniform Yield Test | Laje-Novo Horizonte2 | Bahia |
| 2021.ERU.NH3 | 2021 | Uniform Yield Test | Laje-Novo Horizonte3 | Bahia |
| 2021.ERU.NH4 | 2021 | Uniform Yield Test | Laje-Novo Horizonte4 | Bahia |
| 2021.ERU.AL | 2021 | Uniform Yield Test | Alagoinhas | Bahia |
| 2021.ERU.RIOS | 2021 | Uniform Yield Test | Entre Rios | Bahia |
| 2021.ERU.ALC | 2021 | Uniform Yield Test | Alcobaça | Bahia |
| 2021.ERU.ITAM | 2021 | Uniform Yield Test | Itamarajú | Bahia |
| 2021.ERU.NH1 | 2021 | Uniform Yield Test | Laje-Novo Horizonte1 | Bahia |
| 2021.ERU.NH2 | 2021 | Uniform Yield Test | Laje-Novo Horizonte2 | Bahia |
| 2021.ERU.UFGD | 2021 | Uniform Yield Test | Dourados-UFGD | Mato Grosso do Sul |
| 2021.EC.GS.UFRB | 2021 | Clonal evaluation trial | Cruz das Almas-UFRB | Bahia |
