# Supplementary material for: Genotype x environment interaction in cassava multi-environment trials via analytic factor
Source: PLoS One. 2024 Dec 9;19(12):e0315370. doi: 10.1371/journal.pone.0315370 (PMC11627386; doi:10.1371/journal.pone.0315370)
Supplement: S2 Table — (DOCX) [file pone.0315370.s009.docx]

**Table S2.** Summary of the joint maximum likelihood ratio test analysis of 22 cassava genotypes evaluated in 57, 56, 53 and 59 environments for fresh root yield (FRY), shoot yield (ShY), dry root yield (DRY) and dry matter content in roots (DMC), respectively.

| **Variables** | **model** | **npar** | **logLik** | **AIC** | **LRT** | **Df** | **Pr(>Chisq)** |
| --- | --- | --- | --- | --- | --- | --- | --- |
| FRY | GEN | 5 | -9367.46 | 18744.91 | 270.00 | 1 | 1.13377e-60 |
| FRY | REP(ENV) | 5 | -9267.66 | 18545.33 | 70.41 | 1 | 4.80625e-17 |
| FRY | ENV | 5 | -9355.82 | 18721.64 | 246.73 | 1 | 1.34008e-55 |
| FRY | GEN:ENV | 5 | -9576.62 | 19163.24 | 688.33 | 1 | 1.0305e-151 |
| ShY | GEN | 5 | -9258.74 | 18527.5 | 316.58 | 1 | 8.0442E-71 |
| ShY | REP(ENV) | 5 | -9151.91 | 18313.83 | 102.91 | 1 | 3.50117E-24 |
| ShY | ENV | 5 | -9232.60 | 18475.22 | 264.30 | 1 | 1.98073E-59 |
| ShY | GEN:ENV | 5 | -9401.71 | 18813.43 | 602.51 | 1 | 4.7462E-133 |
| DMC | GEN | 5 | -4952.89 | 9915.79 | 575.92 | 1 | 2.8912e-127 |
| DMC | REP(ENV) | 5 | -4730.22 | 9470.43 | 146.56 | 1 | 3.0835e-30 |
| DMC | ENV | 5 | -4802.22 | 9614.43 | 283.56 | 1 | 1.14933e-61 |
| DMC | GEN:ENV | 5 | -4871.55 | 9753.10 | 445.23 | 1 | 7.25776e-92 |
| DRY | GEN | 5 | -5945.53 | 11901.07 | 265.73 | 1 | 9.69241e-60 |
| DRY | REP(ENV) | 5 | -5843.15 | 11696.29 | 33.95 | 1 | 5.85862e-15 |
| DRY | ENV | 5 | -5939.48 | 11888.96 | 273.61 | 1 | 4.23452e-57 |
| DRY | GEN:ENV | 5 | -6140.97 | 12291.94 | 645.59 | 1 | 8.2316e-145 |
