# Supplementary material for: Genotype x environment interaction in cassava multi-environment trials via analytic factor
Source: PLoS One. 2024 Dec 9;19(12):e0315370. doi: 10.1371/journal.pone.0315370 (PMC11627386; doi:10.1371/journal.pone.0315370)
Supplement: S3 Table — (DOCX) [file pone.0315370.s010.docx]

**Table S3.** Summary of genetic parameters of the joint analysis for fresh root yield of 22 cassava genotypes evaluated in 57, 56, 53 and 59 environments for fresh root yield (FRY), shoot yield (ShY), dry root yield (DRY) and dry matter content in roots (DMC), respectively.

| Parameters | FRY | ShY | DRY | DMC |
| --- | --- | --- | --- | --- |
| $\sigma_{p}^{2}$ | 103.50 | 110.30 | 10.74 | 7.22 |
| $H_{c}^{2}$ | 0.20 | 0.15 | 0.18 | 0.31 |
| $GEIr2$ | 0.30 | 0.26 | 0.30 | 0.19 |
| $h_{mg}^{2}$ | 0.98 | 0.98 | 0.98 | 0.99 |
| $Ac$ | 0.99 | 0.99 | 0.99 | 1.00 |
| $rge$ | 0.44 | 0.37 | 0.43 | 0.40 |
| $CVg$ | 19.14 | 18.25 | 18.54 | 4.20 |
| $CVr$ | 21.18 | 23.84 | 21.41 | 3.14 |
| $CV ratio$ | 0.90 | 0.81 | 0.91 | 1.34 |

$\sigma_{p}^{2}$: phenotypic variance, $H_{c}^{2}$: broad-sense heritability, $h_{mg}^{2}$: plot-based heritability, $Ac$: selection precision, $rge$: environment correlation, $CVg$: coefficient of genotypic variation, $CVr:$ coefficient of residual variation and $CV ratio:$ ratio between the coefficient of genotypic and residual variation.
