# Supplementary material for: Genotype x environment interaction in cassava multi-environment trials via analytic factor
Source: PLoS One. 2024 Dec 9;19(12):e0315370. doi: 10.1371/journal.pone.0315370 (PMC11627386; doi:10.1371/journal.pone.0315370)
Supplement: S4 Table — (DOCX) [file pone.0315370.s011.docx]

**Table S4.** Selected genotypes based on predicted means (BLUP), and slope of the regression ($\beta_{1}$) of the latent regression of the analytic model after varimax rotation.

| Genotypes | Fresh root yield (t ha^-1^) | ${FA}_{1}$ | ${FA}_{2}$ | ${FA}_{3}$ | ${FA}_{4}$ |
| --- | --- | --- | --- | --- | --- |
| BR11-34-41 | 29.21 | -1.02 | 1.06 | 0.35 | **-2.27** |
| BR11-34-45 | 26.44 | 0.06 | -0.61 | -0.90 | -1.07 |
| BR11-34-64 | 28.24 | 0.72 | -0.87 | 0.35 | -0.54 |
| BR11-34-69 | 33.65 | **1.49** | 0.35 | **2.37** | 0.14 |
| BRS Novo Horizonte | 27.69 | 0.73 | 0.69 | 0.97 | 0.51 |
| BRS Poti Branca | 24.21 | -0.40 | **1.52** | 0.44 | 0.09 |
| Cigana Preta | 19.18 | -0.32 | -0.33 | -0.37 | 0.68 |
| Corrente | 20.97 | -1.60 | 0.25 | **1.33** | -0.56 |
| Genotypes | Shoot yield (t ha^-1^) | ${FA}_{1}$ | ${FA}_{2}$ | ${FA}_{3}$ | ${FA}_{4}$ |
| BR11-34-41 | 25.85 | **1.48** | **1.66** | **1.91** | -1.77 |
| BR11-34-45 | 25.80 | 1.08 | 0.92 | 1.11 | -1.07 |
| BR11-34-64 | 23.44 | -1.38 | -0.24 | **1.65** | 0.60 |
| BR11-34-69 | 22.70 | **2.05** | **1.03** | **1.52** | **-2.84** |
| BRS Novo Horizonte | 26.58 | 0.99 | -0.34 | 0.37 | 0.26 |
| BRS Poti Branca | 26.86 | 0.23 | **2.55** | 0.24 | -0.16 |
| Cigana Preta | 22.04 | 0.20 | -0.85 | **1.35** | -0.58 |
| Corrente | 20.32 | -1.03 | -1.63 | -1.33 | **2.15** |
| Genotypes | Dry root yield (t ha^-1^) | ${FA}_{1}$ | ${FA}_{2}$ | ${FA}_{3}$ | ${FA}_{4}$ |
| BR11-34-41 | 9.36 | **1.30** | 0.84 | 0.20 | 0.20 |
| BR11-34-45 | 9.17 | 1.10 | **1.41** | -0.40 | 0.85 |
| BR11-34-64 | 8.23 | 0.68 | 1.08 | -0.69 | 0.83 |
| BR11-34-69 | 9.32 | **1.61** | 1.11 | -0.08 | 0.08 |
| BRS Novo Horizonte | 9.23 | -0.19 | -0.66 | -0.08 | 1.75 |
| BRS Poti Branca | 7.52 | 0.32 | -1.04 | -0.32 | -0.55 |
| Cigana Preta | 6.08 | **-1.32** | -0.47 | 0.90 | -0.84 |
| Corrente | 7.14 | **-1.92** | -0.15 | -0.30 | **2.30** |
| Genotypes | Dry matter content (%) | ${FA}_{1}$ | ${FA}_{2}$ | ${FA}_{3}$ | ${FA}_{4}$ |
| BR11-34-41 | 33.41 | **-2.31** | -0.23 | -0.21 | 0.59 |
| BR11-34-45 | 36.14 | -0.59 | -0.76 | 0.73 | 0.24 |
| BR11-34-64 | 33.94 | -1.79 | -1.03 | -0.53 | 0.56 |
| BR11-34-69 | 33.23 | -1.02 | -0.30 | -1.48 | 0.32 |
| BRS Novo Horizonte | 38.04 | **1.18** | 0.34 | **1.30** | -0.60 |
| BRS Poti Branca | 34.63 | -0.26 | 0.51 | 0.05 | 1.13 |
| Cigana Preta | 36.01 | 0.39 | -0.10 | 0.01 | -0.51 |
| Corrente | 36.57 | 1.08 | 0.78 | 0.39 | -1.18 |

*The angle coefficients with the greatest magnitudes for the selected genotypes are highlighted in bold for all four analytical factor models
