# Supplementary material for: Genotype x environment interaction in cassava multi-environment trials via analytic factor
Source: PLoS One. 2024 Dec 9;19(12):e0315370. doi: 10.1371/journal.pone.0315370 (PMC11627386; doi:10.1371/journal.pone.0315370)
Supplement: S5 Table — (DOCX) [file pone.0315370.s012.docx]

**Table S5.** Analysis of variance by the additive main effects and multiplicative interaction (AMMI) for fresh root yield, shoot yield, dry root yield, and dry matter content in roots, evaluated in 22 cassava genotypes in multi-environment trials.

| Fresh root yield (FRY) | | | | | |  | Shoot yield (ShY) | | | | |
| --- | --- | --- | --- | --- | --- | --- | --- | --- | --- | --- | --- |
| Source | Df | Mean sq | F-value | Pr(>F) | Accumulated |  | Df | Mean sq | F-value | Pr(>F) | Accumulated |
| ENV | 56 | 1664.2 | 30.0 | 0.0 | - |  | 55.0 | 2263.8 | 32.5 | 0.0 | - |
| REP(ENV) | 211 | 55.5 | 2.2 | 0.0 | - |  | 192.0 | 69.8 | 2.7 | 0.0 | - |
| GEN | 21 | 2815.7 | 113.9 | 0.0 | - |  | 21.0 | 3159.0 | 120.2 | 0.0 | - |
| GEN:ENV | 615 | 106.2 | 4.3 | 0.0 | - |  | 633.0 | 96.3 | 3.7 | 0.0 | - |
| PC1 | 76 | 918.8 | 37.2 | 0.0 | 13.2 |  | 75.0 | 1280.3 | 48.7 | 0.0 | 19.5 |
| PC2 | 74 | 925.1 | 37.4 | 0.0 | 26.1 |  | 73.0 | 876.2 | 33.3 | 0.0 | 32.5 |
| PC3 | 72 | 875.4 | 35.4 | 0.0 | 38.0 |  | 71.0 | 859.6 | 32.7 | 0.0 | 44.9 |
| PC4 | 70 | 704.1 | 28.5 | 0.0 | 47.3 |  | 69.0 | 667.2 | 25.4 | 0.0 | 54.3 |
| PC5 | 68 | 671.5 | 27.2 | 0.0 | 56.0 |  | 67.0 | 572.7 | 21.8 | 0.0 | 62.1 |
| PC6 | 66 | 565.9 | 22.9 | 0.0 | 63.0 |  | 65.0 | 478.4 | 18.2 | 0.0 | 68.4 |
| PC7 | 64 | 485.5 | 19.6 | 0.0 | 68.9 |  | 63.0 | 429.9 | 16.4 | 0.0 | 73.9 |
| PC8 | 62 | 459.1 | 18.6 | 0.0 | 74.3 |  | 61.0 | 325.8 | 12.4 | 0.0 | 78.0 |
| PC9 | 60 | 401.2 | 16.2 | 0.0 | 78.8 |  | 59.0 | 301.1 | 11.5 | 0.0 | 81.6 |
| PC10 | 58 | 373.1 | 15.1 | 0.0 | 82.9 |  | 57.0 | 253.9 | 9.7 | 0.0 | 84.5 |
| PC11 | 56 | 315.2 | 12.7 | 0.0 | 86.2 |  | 55.0 | 258.7 | 9.8 | 0.0 | 87.4 |
| **PC12** | **54** | **294.2** | **11.9** | **0.0** | **89.2** |  | **53.0** | **250.7** | **9.5** | **0.0** | **90.1** |
| PC13 | 52 | 292.5 | 11.8 | 0.0 | 92.1 |  | 51.0 | 235.7 | 9.0 | 0.0 | 92.6 |
| PC14 | 50 | 171.2 | 6.9 | 0.0 | 93.7 |  | 49.0 | 198.5 | 7.6 | 0.0 | 94.5 |
| PC15 | 48 | 154.1 | 6.2 | 0.0 | 95.1 |  | 47.0 | 166.0 | 6.3 | 0.0 | 96.1 |
| PC16 | 46 | 146.0 | 5.9 | 0.0 | 96.4 |  | 45.0 | 114.3 | 4.4 | 0.0 | 97.2 |
| PC17 | 44 | 135.9 | 5.5 | 0.0 | 97.5 |  | 43.0 | 95.5 | 3.6 | 0.0 | 98.0 |
| PC18 | 42 | 108.0 | 4.4 | 0.0 | 98.4 |  | 41.0 | 90.7 | 3.5 | 0.0 | 98.8 |
| PC19 | 40 | 95.9 | 3.9 | 0.0 | 99.1 |  | 39.0 | 70.9 | 2.7 | 0.0 | 99.3 |
| PC20 | 38 | 71.5 | 2.9 | 0.0 | 99.6 |  | 37.0 | 60.7 | 2.3 | 0.0 | 99.8 |
| PC21 | 36 | 57.9 | 2.3 | 0.0 | 100.0 |  | 35.0 | 32.0 | 1.2 | 0.2 | 100.0 |
| Residuals | 1934 | 24.7 | - | - | - |  | 1880.0 | 26.3 | - | - | - |
| Total | 4013 | 201.0 | - | - | - |  | 3936.0 | 204.9 | - | - | - |
| Dry root yield (DRY) | | | | | |  | Dry matter content in roots (DMC) | | | | |
| Source | Df | Mean sq | F-value | Pr(>F) | Accumulated |  | Df | Mean sq | F-value | Pr(>F) | Accumulated |
| ENV | 52 | 169.4 | 39.3 | 0.0 | *-* |  | 58.0 | 124.4 | 33.0 | 0.0 | *-* |
| REP(ENV) | 195 | 4.3 | 1.8 | 0.0 | *-* |  | 196.0 | 3.8 | 3.1 | 0.0 | *-* |
| GEN | 21 | 246.8 | 105.0 | 0.0 | *-* |  | 21.0 | 308.7 | 252.6 | 0.0 | *-* |
| GEN:ENV | 582 | 10.2 | 4.3 | 0.0 | *-* |  | 641.0 | 4.3 | 3.5 | 0.0 | *-* |
| PC1 | 72 | 104.5 | 44.4 | 0.0 | 15.5 |  | 78.0 | 60.6 | 49.6 | 0.0 | 18.2 |
| PC2 | 70 | 89.6 | 38.1 | 0.0 | 28.5 |  | 76.0 | 47.8 | 39.1 | 0.0 | 32.1 |
| PC3 | 68 | 77.4 | 32.9 | 0.0 | 39.3 |  | 74.0 | 41.3 | 33.8 | 0.0 | 43.9 |
| PC4 | 66 | 73.6 | 31.3 | 0.0 | 49.3 |  | 72.0 | 32.5 | 26.6 | 0.0 | 52.9 |
| PC5 | 64 | 63.8 | 27.1 | 0.0 | 57.8 |  | 70.0 | 26.7 | 21.9 | 0.0 | 60.1 |
| PC6 | 62 | 53.8 | 22.9 | 0.0 | 64.6 |  | 68.0 | 23.4 | 19.2 | 0.0 | 66.2 |
| PC7 | 60 | 44.9 | 19.1 | 0.0 | 70.2 |  | 66.0 | 23.4 | 19.1 | 0.0 | 72.1 |
| PC8 | 58 | 43.7 | 18.6 | 0.0 | 75.4 |  | 64.0 | 19.4 | 15.9 | 0.0 | 76.9 |
| PC9 | 56 | 38.9 | 16.5 | 0.0 | 79.9 |  | 62.0 | 16.2 | 13.2 | 0.0 | 80.7 |
| PC10 | 54 | 32.6 | 13.9 | 0.0 | 83.6 |  | 60.0 | 16.1 | 13.2 | 0.0 | 84.5 |
| PC11 | 52 | 31.2 | 13.3 | 0.0 | 86.9 |  | 58.0 | 13.9 | 11.3 | 0.0 | 87.6 |
| **PC12** | **50** | **25.2** | **10.7** | **0.0** | **89.5** |  | **56.0** | **11.3** | **9.2** | **0.0** | **90.0** |
| PC13 | 48 | 20.8 | 8.9 | 0.0 | 91.6 |  | 54.0 | 10.4 | 8.5 | 0.0 | 92.1 |
| PC14 | 46 | 20.0 | 8.5 | 0.0 | 93.5 |  | 52.0 | 9.7 | 8.0 | 0.0 | 94.1 |
| PC15 | 44 | 20.5 | 8.7 | 0.0 | 95.3 |  | 50.0 | 8.6 | 7.0 | 0.0 | 95.7 |
| PC16 | 42 | 14.0 | 6.0 | 0.0 | 96.6 |  | 48.0 | 7.2 | 5.9 | 0.0 | 97.1 |
| PC17 | 40 | 14.1 | 6.0 | 0.0 | 97.7 |  | 46.0 | 6.1 | 5.0 | 0.0 | 98.1 |
| PC18 | 38 | 10.9 | 4.6 | 0.0 | 98.6 |  | 44.0 | 4.4 | 3.6 | 0.0 | 98.9 |
| PC19 | 36 | 8.9 | 3.8 | 0.0 | 99.2 |  | 42.0 | 3.4 | 2.7 | 0.0 | 99.4 |
| PC20 | 34 | 5.8 | 2.5 | 0.0 | 99.6 |  | 40.0 | 2.8 | 2.3 | 0.0 | 99.8 |
| PC21 | 32 | 5.5 | 2.4 | 0.0 | 100.0 |  | 38.0 | 1.0 | 0.9 | 0.7 | 100.0 |
| Residuals | 1720 | 2.4 | *-* | *-* | *-* |  | 1824.0 | 1.2 | *-* | *-* | *-* |
| Total | 3662 | 20.0 | *-* | *-* | *-* |  | 3958.0 | 11.5 | *-* | *-* | *-* |
